# Large Language Models for Chatbot Health Advice Studies: A Systematic Review

**DOI:** 10.1001/jamanetworkopen.2024.57879

**Published:** 2025-02-04

**Authors:** Bright Huo, Amy Boyle, Nana Marfo, Wimonchat Tangamornsuksan, Jeremy P. Steen, Tyler McKechnie, Yung Lee, Julio Mayol, Stavros A. Antoniou, Arun James Thirunavukarasu, Stephanie Sanger, Karim Ramji, Gordon Guyatt

**Affiliations:** 1 Division of General Surgery, Department of Surgery, McMaster University, Hamilton, Ontario, Canada; 2 Michael G. DeGroote School of Medicine, McMaster University, Hamilton, Ontario, Canada; 3 H. Ross University School of Medicine, Miramar, Florida; 4 Department of Health Research Methods, Evidence, and Impact, McMaster University, Hamilton, Ontario, Canada; 5 Institute of Health Policy, Management and Evaluation, University of Toronto, Toronto, Ontario, Canada; 6 Hospital Clinico San Carlos, IdISSC, Universidad Complutense de Madrid, Madrid, Spain; 7 Department of Surgery, Papageorgiou General Hospital, Thessaloniki, Greece; 8 Oxford University Clinical Academic Graduate School, University of Oxford, Oxford, United Kingdom; 9 Health Science Library, Faculty of Health Sciences, McMaster University, Hamilton, Ontario, Canada; 10 Department of Clinical Epidemiology and Biostatistics, McMaster University, Hamilton, Ontario, Canada

## Abstract

**Importance:**

There is much interest in the clinical integration of large language models (LLMs) in health care. Many studies have assessed the ability of LLMs to provide health advice, but the quality of their reporting is uncertain.

**Objective:**

To perform a systematic review to examine the reporting variability among peer-reviewed studies evaluating the performance of generative artificial intelligence (AI)–driven chatbots for summarizing evidence and providing health advice to inform the development of the Chatbot Assessment Reporting Tool (CHART).

**Evidence Review:**

A search of MEDLINE via Ovid, Embase via Elsevier, and Web of Science from inception to October 27, 2023, was conducted with the help of a health sciences librarian to yield 7752 articles. Two reviewers screened articles by title and abstract followed by full-text review to identify primary studies evaluating the clinical accuracy of generative AI-driven chatbots in providing health advice (chatbot health advice studies). Two reviewers then performed data extraction for 137 eligible studies.

**Findings:**

A total of 137 studies were included. Studies examined topics in surgery (55 [40.1%]), medicine (51 [37.2%]), and primary care (13 [9.5%]). Many studies focused on treatment (91 [66.4%]), diagnosis (60 [43.8%]), or disease prevention (29 [21.2%]). Most studies (136 [99.3%]) evaluated inaccessible, closed-source LLMs and did not provide enough information to identify the version of the LLM under evaluation. All studies lacked a sufficient description of LLM characteristics, including temperature, token length, fine-tuning availability, layers, and other details. Most studies (136 [99.3%]) did not describe a prompt engineering phase in their study. The date of LLM querying was reported in 54 (39.4%) studies. Most studies (89 [65.0%]) used subjective means to define the successful performance of the chatbot, while less than one-third addressed the ethical, regulatory, and patient safety implications of the clinical integration of LLMs.

**Conclusions and Relevance:**

In this systematic review of 137 chatbot health advice studies, the reporting quality was heterogeneous and may inform the development of the CHART reporting standards. Ethical, regulatory, and patient safety considerations are crucial as interest grows in the clinical integration of LLMs.

## Key Points

**Question:**

What do studies report when evaluating the performance of large language models (LLMs) providing health advice?

**Findings:**

In this systematic review of 137 articles, 99.3% of the studies assessed closed-source models and did not provide enough information to identify the LLM. Most (64.5%) studies used subjective means as the ground truth to define the successful performance of the LLM, while less than a third addressed the ethical, regulatory, and patient safety implications of clinically integrating LLMs.

**Meaning:**

The findings of this study suggest that the extent of reporting varies considerably among studies evaluating the clinical accuracy of LLMs providing health advice.

## Teaser

This systematic review evaluates the reporting quality of studies on the development and use of chatbot health advice services.

## Introduction

Large language models (LLMs) have expanded the potential for integrating artificial intelligence (AI) in medicine. LLMs are AI systems that are pretrained through a variety of word prediction tasks across enormous volumes of text taken from datasets, books, articles, and internet sources.^1,2^ With additional fine-tuning involving varying amounts of human feedback, LLMs acquire natural language processing (NLP) capabilities and can generate appropriate text outputs using layperson terminology in response to free-text prompts.^3^ Many patients and physicians use the internet to access health information,^4^ and generative AI-driven chatbots present a convenient new way to access answers to medical questions.^5^ Early data suggest that generative AI-driven chatbots can even respond to patients with expressions of empathy.^6^

Patients have reported interest in seeking medical advice from chatbots,^7^ leading to a rapid expansion in literature assessing the ability of chatbots to offer sound health advice to physicians and patients: chatbot health advice studies (CHAS).^8^ This research has investigated the ability of chatbots to summarize evidence and provide health advice about screening, diagnosis, treatment, and disease prevention.^9^ However, chatbots are typically not designed for medical application and currently lack regulation for their use in medicine.^10^ Furthermore, generative AI-driven chatbots present unique risks of hallucinating (ie, confident but incorrect) false results and propagating misleading information and advice.^11^ Thus, it is unsurprising that CHAS report varying levels of chatbot performance, raising concerns about their limitations and risks.^1,12,13^

CHAS also present their own unique problems. Currently, these studies are inconsistent in their design, analysis, and reporting.^14,15,16^ With a lack of standardized reporting, the assessment of the quality of CHAS and the interpretation of their findings remain challenging.^17^ These studies have the potential to provide useful assessment of LLM performance and insight into the patient safety implications of chatbot advice, yet the apparent limitations of the studies themselves necessitate clarifying and standardizing current approaches. In this systematic review, we investigated chatbot selection, query approach, and performance evaluation. The information gathered from this systematic review may contribute to the development of a novel chatbot assessment reporting tool (CHART) to standardize reporting methods in chatbot assessment research.^18^

## Methods

This systematic review adheres to the reporting standards of the Preferred Reporting Items for Systematic Reviews and Meta-Analyses (PRISMA) reporting guideline.^19^ In this study, we refer to LLMs, generative AI-driven chatbots, and chatbots synonymously. Our protocol was prospectively registered on Open Sciences Framework.^20^

### Search Strategy and Selection Criteria

We set out to characterize the approach of studies that assess the performance of generative AI-driven chatbots for summarizing evidence and providing health advice. To capture all relevant studies, an academic health sciences librarian (S.S.) with expertise in systematic reviews aided the study team in designing a comprehensive search strategy. The detailed literature search syntax can be found in the eAppendix in Supplement 1. Records were retrieved from MEDLINE via Ovid, Embase via Elsevier, and Web of Science on October 27, 2023. The study team completed 2 rounds of screening, first by title and abstract and then by full text. In all cases, 2 independent researchers (W.T. and J.P.S.) conducted screening, with a third independent researcher casting a deciding vote to resolve cases of disagreement. We excluded irrelevant studies and nonprimary studies. The Box illustrates our eligibility criteria.

Box. **Inclusion and Exclusion Criteria**

**Inclusion Criteria**

- Studies assessing the performance of chatbots for summarizing clinical evidence and providing health advice.

**Exclusion Criteria**

- Systematic reviews and literature/narrative reviews.
- Editorials, commentaries, opinions, studies published as correspondence articles, and studies of any type outlining earlier or potential future applications of chatbots.
- Cross-sectional studies assessing opinions of future work for chatbot research.
- Abstracts.

### Inclusion and Exclusion Criteria

Data extraction was conducted with Covidence and aimed to capture basic study details (clinical specialty, topic, intervention, and comparator) in addition to the following: (1) how LLMs are selected for use in CHAS, (2) clinical purposes for which LLMs have been evaluated, (3) methods applied, (4) approach to performance evaluation (ie, summarizing evidence alone or summarizing evidence to inform recommendation), (5) reporting practices used, and (6) the presence of ethical considerations of the clinical application of generative AI-driven chatbots.

### Statistical Analysis

The study team performed data analysis using descriptive statistics. We summarized findings pertaining to the methodologic approach of CHAS using frequencies as counts and percentages including the types of LLMs evaluated, the query strategy, and their performance evaluation. A narrative synthesis with no meta-analysis was planned, due to anticipated heterogeneity in study subjects and designs. We describe the full details related to the literature search and data extraction variables in our study protocol.^20^ The quality of the evidence was not evaluated due to the lack of high-quality, validated risk-of-bias tools available for CHAS.

## Results

### Study Characteristics

The study team reviewed 7752 articles that yielded 137 eligible CHAS (Figure). Table 1 lists all evaluated articles,^6,13,21,22,23,24,25,26,27,28,29,30,31,32,33,34,35,36,37,38,39,40,41,42,43,44,45,46,47,48,49,50,51,52,53,54,55,56,57,58,59,60,61,62,63,64,65,66,67,68,69,70,71,72,73,74,75,76,77,78,79,80,81,82,83,84,85,86,87,88,89,90,91,92,93,94,95,96,97,98,99,100,101,102,103,104,105,106,107,108,109,110,111,112,113,114,115,116,117,118,119,120,121,122,123,124,125,126,127,128,129,130,131,132,133,134,135,136,137,138,139,140,141,142,143,144,145,146,147,148,149,150,151,152,153,154,155^ including their relevant specialty and study aims.

**Figure.**
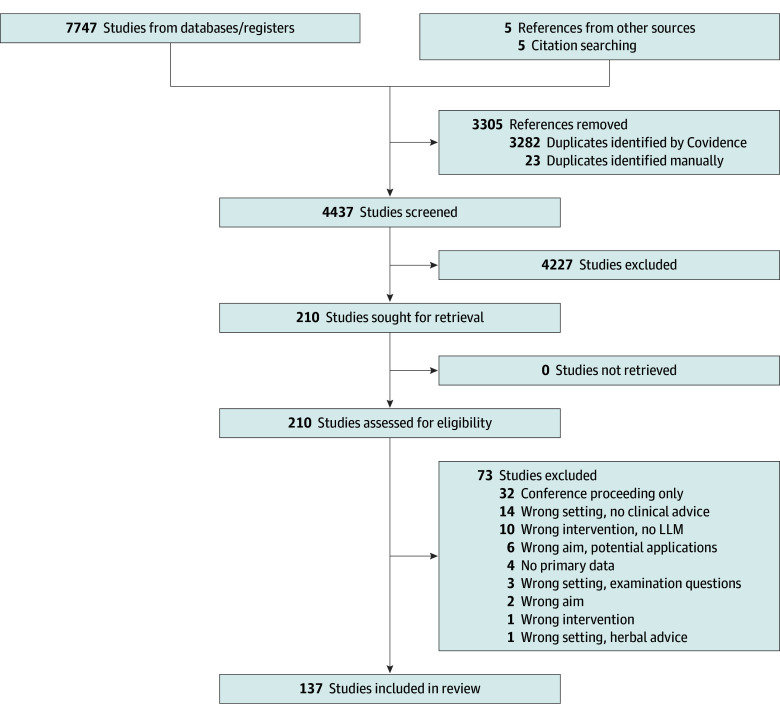
Overview of Literature Search There were no ongoing studies or studies awaiting classification. LLM indicates large language model.

**Table 1.** Study Characteristics

Study	Topic specialty	Health advice aim
Al-Ashwal et al,^21^ 2023	Medicine	Treatment
Alessandri-Bonetti et al,^22^ 2024	Medicine	Diagnosis; treatment; general information
Ali et al,^23^ 2023	Surgery	Treatment; general information
Ali et al,^24^ 2023	Medicine	General information
Altamimi et al,^25^ 2023	Primary care (FM, EM)	Disease prevention; treatment; general information
Athavale et al,^26^ 2023	Medicine	Disease prevention; treatment; general information
Ayers et al,^27^ 2023	Primary care (FM, EM)	Disease prevention; treatment; general information
Ayers et al,^6^ 2023	Primary care (FM, EM)	Disease prevention; diagnosis; treatment; general information
Ayoub et al,^28^ 2023	Medicine	Differential diagnosis; diagnosis; treatment
Ayoub et al,^29^ 2023	Surgery	Treatment; general information
Balel et al,^30^ 2023	Surgery	Screening; diagnosis; treatment; general information
Bellinger et al,^31^ 2024	Surgery	Diagnosis; treatment; general information
Benirschke et al,^32^ 2024	Pathology	General information
Bernstein et al,^33^ 2023	Medicine	Treatment; general information
Biswas et al,^34^ 2023	Primary care (FM, EM)	Disease prevention; screening; treatment; general information
Caglar et al,^35^ 2024	Surgery	Treatment; general information
Cakir et al,^36^ 2024	Medicine	Diagnosis; treatment; general information
Campbell et al,^37^ 2023	Medicine	Diagnosis; treatment; general information
Caruccio et al,^38^ 2024	Primary care (FM, EM)	Diagnosis
Chee et al,^39^ 2023	Medicine	Differential diagnosis; diagnosis; treatment
Chen et al,^40^ 2023	Medicine	Diagnosis; treatment
Chervenak et al,^41^ 2023	Medicine	Disease prevention; treatment; general information
Chiesa-Estomba et al,^42^ 2024	Surgery	Treatment
Chowdhury et al,^43^ 2023	Surgery	General information
Cocci et al,^44^ 2024	Surgery	Diagnosis; treatment; general information
Coskun et al,^45^ 2024	Medicine	Treatment; general information
Coskun et al,^46^ 2023	Primary care (FM, EM)	Disease prevention; screening; diagnosis; treatment; general information
Cox et al,^47^ 2023	Surgery	General information
Davis et al,^13^ 2023	Surgery	Diagnosis; treatment; general information
Deiana et al,^48^2023	Primary care (FM, EM)	Disease prevention; general information
Dubin et al,^49^ 2023	Surgery	Treatment; general information
Dwyer et al,^50^ 2023	Surgery	Treatment; general information
Emile et al,^51^ 2023	Surgery	Disease prevention; screening; diagnosis; treatment; general information
Endo et al,^52^ 2023	Surgery	General information
Farhat et al,^53^ 2024	Psychiatry	Treatment; general information
Franco D’Souza et al,^54^ 2023	Psychiatry	Differential diagnosis; diagnosis; treatment; general information
Fraser et al,^55^ 2023	Primary care (FM, EM)	Diagnosis
Gabriel et al,^56^ 2023	Surgery	General information
Galido et al,^57^ 2023	Psychiatry	Differential diagnosis; treatment
Gebrael et al,^58^ 2023	Primary care (FM, EM)	Differential diagnosis; diagnosis; treatment
Goodman et al,^59^ 2023	Medicine	Disease prevention; screening; differential diagnosis; diagnosis; treatment; general information
Gordon et al,^60^ 2024	Radiology	General information
Gracias et al,^61^ 2024	Surgery	Disease prevention; treatment; general information
Gravel et al,^62^ 2023	Medicine	Treatment; general information
Gwak et al,^63^ 2023	Primary care (FM, EM)	Disease prevention; differential diagnosis; treatment; general information
Haemmerli et al,^64^ 2023	Medicine	Diagnosis; treatment
Harskamp et al,^65^ 2024	Medicine	Diagnosis; treatment; general information
Haver et al,^66^ 2023	Radiology	Disease prevention; screening
Haver et al,^67^ 2023	Radiology	Disease prevention; screening; general information
Hirosawa et al,^68^ 2023	Medicine	Differential diagnosis; diagnosis
Hirosawa et al,^69^ 2023	Medicine	Differential diagnosis
Hirosawa et al,^70^ 2023	Medicine	Differential diagnosis; diagnosis
Hopkins et al,^71^ 2023	Medicine	General information
Hristidis et al,^72^ 2023	Medicine	General information
Hurley et al,^73^ 2024	Surgery	Treatment; general information
Janopaul-Naylor et al,^74^ 2024	Medicine	Prognosis, treatment
Johnson et al,^75^ 2023	Mixed	Treatment
Kao et al,^76^ 2023	Medicine	Differential diagnosis; diagnosis
Kataoka et al,^77^ 2021	Medicine	Treatment; general information
King et al,^78^ 2024	Medicine	Treatment; general information
Koh et al,^79^ 2023	Medicine	General information
Krusche et al,^80^ 2024	Medicine	Differential diagnosis; diagnosis
Kuroiwa et al,^81^ 2023	Surgery	Differential diagnosis; diagnosis; general information
Kusunose et al,^82^ 2023	Medicine	General information
Lahat et al,^83^ 2023	Medicine	Diagnosis; treatment; general information
Lam et al,^84^ 2024	Medicine	Treatment; general information
Lechien et al,^85^ 2024	Surgery	Differential diagnosis; diagnosis; treatment
Lee et al,^86^ 2023	Surgery	General information
Levartovsky et al,^87^ 2023	Medicine	Treatment
Levkovich et al,^88^ 2023	Psychiatry	Treatment
Levkovich et al,^89^ 2023	Psychiatry	Disease prevention; diagnosis
Li et al,^90^ 2023	Surgery	Screening; differential diagnosis; diagnosis; treatment
Li et al,^91^ 2023	Surgery	General information
Lim et al,^92^ 2023	Surgery	Disease prevention; diagnosis; treatment; general information
Lim et al,^93^ 20244	Medicine	Screening; general information
Liu et al,^94^ 2023	Medicine	Screening; general information
Liu et al,^95^ 2023	Surgery	Treatment
Liu et al,^96^ 2023	Surgery	General information
Long et al,^97^ 2024	Primary care (FM, EM)	General information
Lower et al,^98^ 2023	Surgery	Diagnosis; treatment; general information
Luykx et al,^99^ 2023	Surgery	Treatment; general information
Lyons et al,^100^ 2024	Psychiatry	Diagnosis; treatment; general information
Maillard et al,^101^ 2024	Surgery	Differential diagnosis; diagnosis
Manolitsis et al,^102^ 2023	Medicine	Diagnosis; treatment
Mehnen et al,^103^ 2023	Surgery	Disease prevention; treatment; general information
Mesnier et al,^104^ 2023	Medicine	Differential diagnosis; diagnosis
Mika et al,^105^ 2023	Medicine	Diagnosis; treatment; general information
Mishra et al,^106^ 2023	Surgery	Treatment; general information
Momenaei et al,^107^ 2023	Surgery	Treatment
Moshirfar et al,^108^ 2023	Surgery	Diagnosis; treatment; general information
Musheyev et al,^109^ 2024	Surgery	General information
Nastasi et al,^110^ 2023	Medicine	Disease prevention; treatment; general information
Nielsen et al,^111^ 2023	Medicine	Differential diagnosis; diagnosis; treatment; general information
O’Hagan et al,^112^ 2023	Surgery	Diagnosis; treatment; general information
Padovan et al,^113^ 2024	Medicine	Diagnosis; treatment; general information
Pan et al,^114^ 2023	Medicine	General information
Potapenko et al,^115^ 2023	Medicine	General information
Potapenko et al,^116^ 2023	Surgery	Disease prevention; treatment; general information
Qu et al,^117^ 2023	Surgery	Disease prevention; treatment; general information
Rahsepar et al,^118^ 2023	Surgery	Differential diagnosis; diagnosis; treatment
Rao et al,^119^ 2023	Radiology	Disease prevention; screening; general information
Rao et al,^120^ 2023	Medicine	Differential diagnosis; diagnosis; treatment
Rasmussen et al,^121^ 2023	Medicine	Differential diagnosis; diagnosis; treatment
Rau et al,^122^ 2023	Radiology	Screening; general information
Rizwan et al,^123^ 2023	Surgery	Disease prevention; treatment; general information
Rogasch et al,^124^ 2023	Radiology	Diagnosis
Rojas-Carabali et al,^125^ 2024	Medicine	Diagnosis; treatment
Rosen et al,^126^ 2023	Radiology	Treatment
Rosen et al,^127^ 2024	Surgery	Differential diagnosis; diagnosis; treatment
Samaan et al,^128^ 2023	Primary care (FM, EM)	Diagnosis
Samaan et al,^129^ 2023	Radiology	Diagnosis
Schulte et al,^130^ 2023	Medicine	Disease prevention; treatment; general information
Seth et al,^131^ 2023	Surgery	General information
Seth et al,^132^ 2023	Surgery	Diagnosis; treatment
Seth et al,^133^ 2023	Surgery	General information
Sezgin et al,^134^ 2023	Surgery	Treatment; general information
Shao et al,^135^ 2023	Surgery	Diagnosis; treatment
Sorin et al,^136^ 2023	Psychiatry	General information
Stevenson et al,^137^ 2024	Surgery	Disease prevention; diagnosis; treatment; general information
Stroop et al,^138^ 2024	Medicine	Treatment
Suresh et al,^139^ 2023	Medicine	Diagnosis; general information
Szczesniewski et al,^140^ 2023	Surgery	Diagnosis; treatment; general information
Vaira et al,^141^ 2024	Surgery	Diagnosis; treatment; general information
Van Bulck et al,^142^ 2024	Surgery	Treatment; general information
Wagner et al,^143^ 2024	Surgery	Disease prevention; diagnosis; treatment; general information
Walker et al,^144^ 2023	Medicine	Disease prevention; treatment
Wang et al,^145^ 2023	Radiology	Differential diagnosis; diagnosis; treatment; general information
Wang et al,^146^ 2023	Surgery	Treatment; general information
Xie et al,^147^ 2023	Medicine	Treatment
Yeo et al,^148^ 2023	Surgery	Treatment; general information
Yildiz et al,^149^ 2023	Surgery	General information
Yun et al,^150^ 2023	Medicine	Disease prevention; diagnosis; treatment; general information
Zhou et al,^151^ 2023	Medicine	Diagnosis; treatment
Zhou et al,^152^ 2023	Surgery	Treatment; general information
Zhou et al,^153^ 2024	Surgery	Treatment
Zhu et al,^154^ 2023	Medicine	Differential diagnosis; diagnosis; treatment; general information
Zúñiga Salazar et al,^155^ 2023	Surgery	Diagnosis; treatment; general information

Abbreviations: EM, emergency medicine; FM, family medicine.

Overall, of the 137 articles reviewed, 51 (37.2%) studies queried LLMs for advice relevant for medical topics, 55 (40.1%) on surgical topics, 13 (9.5%) on primary care topics, 9 (6.6%) on radiology topics, and 7 (5.1%) on psychiatry topics (Table 1). Articles most commonly examined advice regarding treatment (91 [66.4%]), diagnosis (60 [43.8%]), and differential diagnosis (23 [16.8%]). Overall, 16 studies (11.7%) explained why the specific LLMs were selected to be studied. Almost all studies (135 [99.3%]) assessed the ability of the OpenAI Chat Generative Pretrained Transformer (ChatGPT) to provide health advice, and 136 of the LLMs studied (99.3%) were inaccessible (proprietary/closed-source) models. A single study (0.7%) fully described the version of the LLM under evaluation, and no studies described the characteristics of the LLM in comprehensive detail including temperature (a parameter to control the randomness of LLM output), token length, fine-tuning availability, penalties, add-on availability, language, and layers related to the LLM being studied. More than one-quarter (36 [26.3%]) of the studies described none of the characteristics of the LLM being examined (Table 2). Sources used to develop prompts included expert opinion (43 [31.4%]), professional society websites (28 [20.4%]), and clinical practice guidelines (24 [17.5%]).

**Table 2.** Study Methods and Performance Evaluation Variables for Extraction

Variable	No. (%) (N = 137)
Specialty	
Medicine	51 (37.2)^21,22,24,26,28,33,35,37,39,40,41,45,59,62,64,65,68,69,70,71,72,74,76,77,78,79,80,82,83,84,87,93,101,103,104,109,110,112,113,114,119,123,128,130,136,137,142,145,148,149,152^
Mixed^a^	1 (0.7)^75^
Pathology	1 (0.7)^32^
Primary care^b^	13 (9.5)^6,25,27,34,38,46,48,55,58,63,96,126,155^
Psychiatry	7 (5.1)^53,54,57,88,89,99,134^
Radiology	9 (6.6)^60,66,67,118,120,122,124,127,143^
Surgery	55 (40.1)^13,23,30,31,36,42,43,44,47,49,50,51,52,56,61,73,81,85,86,90,91,92,94,95,97,98,100,102,105,106,107,108,111,115,117,121,125,129,131,132,133,135,138,139,140,141,144,146,147,150,151,153,154^
Aim	
Disease prevention	29 (21.2)^6,25,26,27,34,41,46,48,51,59,61,63,66,67,89,92,102,110,115,116,118,121,128,135,141,142,148,154,155^
Screening	12 (8.8)^30,34,46,51,59,66,67,90,93,118,120,154^
Differential diagnosis	23 (16.8)^28,39,54,57,58,59,63,68,69,70,76,80,81,85,90,100,103,110,117,119,125,143,152^
Diagnosis	60 (43.8)^6,13,22,28,30,31,35,37,38,39,40,44,46,51,54,55,58,59,64,65,68,70,76,80,81,83,85,89,90,92,97,99,100,101,103,104,107,110,111,112,117,119,122,123,125,126,127,130,133,135,137,138,139,141,143,148,149,152,153,155^
Treatment	91 (66.4)^6,13,21,22,23,25,26,27,28,30,31,33,34,35,36,37,39,40,41,42,44,45,46,49,50,51,53,54,57,58,59,61,62,63,64,65,73,75,77,78,83,84,85,87,88,92,94,97,98,99,101,102,104,105,106,107,110,111,112,115,116,117,119,121,123,124,125,128,130,132,133,135,136,138,139,140,141,142,143,144,145,146,148,149,150,151,152,153,154,155^
General information	85 (62.0)^6,22,23,24,25,26,30,31,33,34,35,36,37,41,44,45,46,47,48,49,50,51,52,53,54,59,60,61,62,63,65,67,71,72,73,77,78,79,81,83,84,86,91,92,93,95,96,97,98,99,102,104,105,107,109,110,111,112,113,114,115,116,118,120,121,128,131,132,134,135,138,139,140,141,143,144,146,147,148,150,152,153^
Selection of generative AI-driven chatbots	
Follow-up on earlier study	8 (5.8)^55,66,67,80,120,143,149,152^
Location/availability	4 (2.9)^25,31,91,118^
Accessibility (open vs closed)	4 (2.9)^33,37,123,147^
Not described	121 (88.3)^6,13,21,22,24,27,28,29,30,33,34,35,36,38,39,40,41,42,43,44,45,46,47,48,49,51,52,53,54,56,57,58,59,60,61,62,63,64,65,68,69,70,71,72,73,74,75,76,78,79,81,82,83,84,85,86,87,88,89,90,92,93,94,95,96,97,98,99,100,101,102,103,104,105,106,107,108,110,111,112,113,114,115,116,117,118,119,121,122,125,126,127,128,129,130,131,132,133,134,135,136,137,138,139,140,141,142,144,145,146,148,150,151,152,153,155^
LLM	
ChatGPT	135 (98.5)^6,13,21,22,23,24,25,26,27,28,29,30,31,33,34,35,36,37,38,39,40,41,42,43,44,45,46,47,48,49,50,51,52,53,54,55,56,57,58,59,60,61,62,63,64,65,66,67,68,69,71,72,73,74,75,76,77,78,79,80,81,82,83,84,85,86,87,88,89,90,91,92,93,94,95,96,98,99,100,101,102,103,104,105,106,107,108,109,110,111,112,113,114,115,116,117,118,119,120,121,122,123,124,125,126,127,128,129,130,131,132,133,134,135,136,137,138,139,140,141,142,143,144,146,147,148,149,150,151,153,154^
Google Bard	11 (8.0)^21,22,38,45,70,92,118,132,134,137,155^
Bing Chat	8 (5.8)^74,100,109,114^
Novel LLM	4 (2.9)^50,77,97,152^
Other LLM	4 (2.9)^26,114,145,154^
Accessibility	
Accessible (open)	2 (1.5)^50,77^
Inaccessible (closed)	136 (99.3)^6,13,21,22,23,24,25,26,27,28,29,30,31,33,34,35,36,37,38,39,40,41,42,43,44,45,46,47,48,49,51,52,53,54,55,56,57,58,59,60,61,62,63,64,65,66,67,68,69,70,71,72,73,74,75,76,78,79,80,81,82,83,84,85,86,87,88,89,90,91,92,93,94,95,96,97,98,99,100,101,102,103,104,105,106,107,108,109,110,111,112,113,114,115,116,117,118,119,120,121,122,123,124,125,126,127,128,129,130,131,132,133,134,135,136,137,138,139,140,141,142,143,144,145,146,147,148,149,150,151,152,153,154,155^
LLM characteristics	
Temperature	2 (1.5)^82,145^
Token length	3 (2.2)^97,102,122^
Fine-tuning availability	3 (2.2)^44,145,152^
Penalties	0 (0.0)
Add-on availability	0 (0.0)
Date accessed/trained	64 (46.7)^6,13,22,24,27,30,31,33,34,35,36,37,41,42,43,44,45,46,50,56,59,64,66,67,68,69,70,71,73,74,80,81,82,84,86,88,89,100,105,106,107,109,110,111,112,114,115,117,118,119,120,124,128,129,130,134,136,137,140,141,142,143,144,148,152,153^
LLM version^c^	1 (0.7)^145^
Language	10 (7.3)^28,65,69,82,128,135,136,137,140,149^
Layers	0 (0.0)
None described	36(26.3)^21,29,39,47,49,51,52,53,57,60,62,72,76,77,78,79,83,90,91,92,94,95,96,103,104,110,113,116,121,123,126,127,133,138,147,150,151^
Sources of prompts	
Expert opinion	43 (31.4)^21,26,27,28,30,45,50,59,60,61,64,68,70,72,75,76,77,81,98,101,104,107,108,110,113,115,117,118,121,123,124,131,132,133,135,137,138,141,142,149,150,154^
Guidelines	24 (17.5)^25,29,41,47,59,65,75,82,90,91,93,94,102,120,122,125,130,135,139,145,146,147,154^
Professional society website	28 (20.4)^22,24,25,33,34,35,36,46,48,51,56,67,69,74,78,81,84,92,97,103,122,128,129,134,146,147,148,154^
Social media (patient questions)	8 (5.8)^6,35,36,55,78,128,129,155^
Textbook	4 (2.9)^54,117,119,131^
Website (non–evidence-based)	12 (8.8)^24,31,33,65,72,86,95,105,109,114,119,153^
Websites/forums (patient questions)	12 (8.8)^6,13,31,33,36,41,49,73,83,105,117,128^
None described	37 (27.0)^23,37,38,39,40,42,43,44,52,53,57,58,62,63,66,71,79,80,85,87,88,89,96,99,100,106,111,112,116,126,127,136,140,143,144,151,152^
Prompt engineering/testing	
Yes	3 (2.2)^44,81,153^
No	136 (99.3)^6,13,21,22,23,24,25,26,27,28,29,30,31,33,34,35,36,37,38,39,40,41,42,43,45,46,47,48,49,50,51,52,53,54,55,56,57,58,59,60,61,62,63,64,65,66,67,68,69,70,71,72,73,74,75,76,77,78,79,80,82,83,84,85,86,87,88,89,90,91,92,93,94,95,96,97,98,99,100,101,102,103,104,105,106,107,108,109,110,111,112,113,114,115,116,117,118,119,120,121,122,123,124,125,126,127,128,129,130,131,132,133,134,135,136,137,138,139,140,141,142,143,144,145,146,147,148,149,150,151,152,154,155^
Standardized prompts	
Yes	20 (14.6)^21,22,24,29,31,33,37,43,47,60,62,81,92,100,106,114,117,125,130,153^
No	119 (86.9)^6,13,23,25,26,27,28,30,33,34,35,36,38,39,40,41,42,44,45,46,48,49,50,51,52,53,54,55,56,57,58,59,61,63,64,65,67,68,69,70,71,72,73,74,75,76,77,78,79,80,82,83,84,85,86,87,88,89,90,91,93,94,95,96,97,98,99,101,102,103,104,105,107,108,109,110,111,112,113,115,116,118,119,120,121,122,123,124,126,127,128,129,131,132,133,134,135,136,137,138,139,140,141,142,143,144,145,146,147,148,149,150,151,152,154,155^
Structures of prompts	
Narrative	136 (99.3)^6,13,22,23,24,25,26,27,28,29,30,31,33,34,35,36,37,38,39,40,41,42,43,44,45,46,47,48,49,50,51,52,53,55,56,57,58,59,60,61,62,64,65,66,67,68,69,70,71,72,73,74,75,76,77,78,79,80,81,82,83,84,85,86,87,88,89,90,91,92,93,94,95,96,98,99,100,101,102,103,104,105,106,107,108,109,110,111,112,113,114,115,116,117,118,119,120,121,122,123,124,125,126,127,128,129,130,131,132,133,134,135,136,137,138,139,140,141,142,143,144,145,146,147,148,149,150,151,152,153,154,155^
MCQ	2 (1.5)^21,54^
Mixed narrative plus MCQ	1 (0.7)^97^
Prompt inclusion	
Yes, in-text, partial	17 (12.4)^6,38,43,49,50,52,67,68,69,70,75,97,102,110,134,145,155^
Yes, in-text, full	44 (32.2)^23,25,26,27,37,42,47,48,51,53,56,57,58,60,61,63,65,66,71,73,76,84,87,91,94,95,98,107,112,117,118,123,124,126,127,131,133,135,147,149,150,151,153,154^
Yes, in supplementary file, partial	12 (8.8)^33,41,83,89,93,101,110,114,119,122,136,138,144^
Yes, in supplementary file, full	49(35.7)^13,24,28,29,30,31,33,34,35,36,39,45,46,54,59,62,64,72,74,77,80,81,86,88,90,92,96,99,100,104,105,106,109,111,115,116,119,120,121,125,129,132,137,139,141,142,143,146,148^
No	17 (12.4)^21,22,40,44,55,78,79,82,85,93,103,108,113,128,130,140,152^
Chatbot response inclusion	
Yes, in-text, partial	29 (21.2)^6,23,27,29,38,43,44,49,50,52,66,67,68,69,70,72,75,85,94,97,100,102,103,106,116,118,127,145,155^
Yes, in-text, full	23 (16.8)^25,47,48,53,56,57,58,61,63,65,71,84,87,91,95,98,123,126,131,133,147,149,153^
Yes, in supplementary file, partial	16 (11.7)^33,40,41,77,83,86,89,93,101,107,109,112,114,122,136,144^
Yes, in supplementary file, full	42 (30.7)^13,24,26,30,31,33,34,39,45,46,54,59,62,64,73,74,81,90,92,96,99,104,105,111,115,117,119,120,121,124,125,129,132,134,135,137,139,141,142,146,148^
No	29 (21.7)^21,22,28,35,36,37,42,51,55,60,76,78,79,80,82,88,93,108,110,113,128,130,138,140,143,150,151,152,154^
Query strategy	
Date	54 (39.4)^6,13,22,27,30,33,34,35,42,45,46,47,49,50,52,55,56,58,63,64,66,69,71,72,74,81,82,84,86,88,92,99,105,106,107,108,109,110,111,112,114,117,118,119,124,130,134,136,137,140,141,143,153,154^
Location	5 (3.6)^47,49,50,58,72^
Querier^d^	36 (26.3)^13,24,25,27,31,33,37,41,42,44,47,48,55,58,59,60,62,68,69,70,72,75,81,88,89,100,104,110,114,118,120,129,130,131,135,136^
No. of prompts/windows	32 (23.4)^24,28,30,31,33,37,43,44,48,57,58,64,67,69,78,89,91,103,107,108,109,112,114,115,119,124,125,129,130,143,148^
No. of users	8 (5.8)^41,62,70,104,115,118,120,129^
No. of queries	48 (35.0)^36,46,47,51,53,58,67,69,71,76,78,81,86,88,93,94,97,99,103,105,106,107,114,115,119,121,122,124,132,134,143,146,148^
Use of check queries^e^	3 (2.2)^47,59,115^
Not described	40 (29.2)^21,23,26,29,38,39,40,54,61,65,73,77,79,80,83,85,87,90,95,96,98,101,102,113,116,123,126,127,128,133,138,139,142,144,145,147,149,151,152,155^
Performance evaluation	
No. of evaluators	97 (70.8)^6,13,23,24,26,27,28,29,31,33,34,35,36,37,40,42,43,44,45,46,48,51,52,56,59,60,61,62,64,65,66,67,68,69,70,72,73,74,75,76,78,81,82,83,84,85,86,90,91,92,93,94,95,96,98,99,100,101,104,106,107,110,111,112,114,115,116,118,119,120,121,122,124,125,128,129,131,132,133,134,135,136,137,138,140,141,142,144,145,146,147,148,149,150^
Randomized order	0 (0.0)
Blinding	16 (11.7)^6,27,28,29,31,55,69,72,85,95,99,101,109,112,113,114^
Standardization/training	18 (13.1)^27,28,29,31,33,43,44,60,72,84,90,92,106,110,113,129,134,150^
Not described	39 (28.4)^21,22,25,30,38,39,41,47,49,50,53,54,57,58,63,71,77,79,80,87,88,89,97,102,103,105,108,110,117,123,126,127,130,139,143,151,152,153,154,155^
Performance definition	
Evidence summary	0 (0.0)
Evidence-based	0 (0.0)
Partially evidence-based	0 (0.0)
Evidence summary and recommendations	21 (15.3)^22,40,41,44,51,65,85,90,101,109,110,114,120,122,127,130,132,139,140,145,146^
Evidence-based	4 (2.9)^22,51,85,139^
Partially evidence-based	15 (10.9)^40,44,65,90,109,110,114,120,122,127,130,132,140,144,146^
Traditional textbook	1 (0.7)^54^
Electronic compendium^f^	2 (1.5)^97,119^
Organization website	1 (0.7)^60^
Investigators without reference to source	89 (65.0)^6,13,21,23,24,25,26,27,28,29,30,31,33,34,35,36,42,43,45,46,48,55,56,58,59,61,62,64,66,67,68,69,70,71,72,73,74,75,76,77,78,79,80,81,82,83,84,86,87,89,93,95,96,98,99,100,103,104,105,106,107,111,112,113,115,116,117,118,119,121,124,125,126,128,129,131,133,134,136,137,138,141,142,147,148,149,150,151,152,155^
Investigator panel	3 (2.2)^50,110,123^
Primary article	3 (2.2)^37,88,91^
Not reported	14 (10.2)^38,39,47,49,53,57,63,94,102,108,135,143,144,153,154^
Power calculation	
Yes	3 (2.2)^6,55,68,76,127^
No	134 (97.8)^13,21,22,23,24,25,26,27,28,29,30,31,33,34,35,36,37,38,39,40,41,42,43,44,45,46,47,48,49,50,51,52,53,54,56,57,58,59,60,61,62,63,64,65,66,67,69,70,71,72,73,74,75,77,78,79,80,81,82,83,84,85,86,87,88,89,90,91,92,93,94,95,96,97,98,99,100,101,102,103,104,105,106,107,108,109,110,111,112,113,114,115,116,117,118,119,120,121,122,123,124,125,126,128,129,130,131,132,133,134,135,136,137,138,139,140,141,142,143,144,145,146,147,148,149,150,151,152,153,154,155^
Ethics	45 (32.8)^6,21,25,33,34,38,43,44,46,48,54,55,56,58,59,61,65,72,73,75,78,80,87,88,89,94,98,99,100,102,113,117,122,123,125,126,127,131,132,134,138,141,145,149^
Patient safety	44 (32.8)^26,28,29,30,33,38,40,42,43,47,53,55,56,57,58,60,61,62,78,96,97,98,100,101,102,104,110,113,126,129,131,132,133,134,135,136,137,138,139,141,146,149,153^
Regulation of LLMs	22 (16.1)^13,26,27,38,46,48,58,61,70,71,78,81,82,87,94,104,110,119,120,126,127^
Not reported	61 (44.5)^22,23,24,31,35,36,37,39,41,45,49,50,51,52,63,64,66,67,68,69,74,76,77,79,83,84,85,86,90,91,92,93,95,103,105,106,107,108,109,111,112,114,115,116,118,121,124,128,130,140,142,143,144,147,148,150,151,152,154,155^

Abbreviations: AI, artificial intelligence; Chat GPT, OpenAI Chat Generative Pretrained Transformer; LLM, large language model; MCQ, multiple-choice question.

^a^ Medicine and surgery.

^b^ Family medicine and emergency medicine.

^c^ For studies using ChatGPT, 59 articles reported the model number (3.5 vs 4).

^d^ Individuals who performed the query.

^e^ UpToDate, DynaMed.

^f^ Check queries: repeating prompts/analysis to ensure consistency before submission for publication.

### Performance Evaluation

Table 2 presents details of our study performance evaluation. More than one-quarter (37 [27.0%]) of the studies did not explain the source of their prompts or describe at least one aspect of their query strategy (40 [29.2%]). Few studies noted the date (54 [39.4%]) and location (5 [3.6%]) of their query. Study performance was suboptimal in noting the number of chat windows (32 [23.4%]) and the number of queries (48 [35.0%]). Studies performed poorly (20 [14.6%]) in using standardized prompts to evaluate their LLMs. Overall, 136 articles (99.3%) did not describe a prompt testing/engineering phase, while 93 (67.9%) shared the full transcript of their prompts and 65 (47.4%) included the full transcript of chatbot responses.

With respect to performance evaluation of chatbots, few studies described a standardized evaluation process (18 [13.1%]) or blinding (16 [11.7%]), and many studies did not describe a structured approach to performance evaluation (39 [28.5%]). Rather than using clinical practice guideline recommendations as in 21 studies (15.3%), most investigators evaluated LLMs based on their opinion without a reference standard (89 [65.0%]). Less than one-third of the studies addressed the ethical (45 [32.8%]) and patient safety (44 [32.1%]) implications of the clinical integration of LLMs, while 22 (16.1%) addressed the lack of regulation of LLMs (Table 2).

## Discussion

This systematic review identified large heterogeneity in the methods of CHAS and a need for improved reporting standards. Many studies failed to adequately report the methods of chatbot performance evaluation. Moreover, most studies that described an approach to evaluation relied on anecdotal opinion rather than systematic summaries of available evidence or official guidelines. No study described the characteristics of the LLM under evaluation in sufficient detail to reproduce experiments, and many failed to describe any aspect of the LLM characteristics. Most reports did not include reasons for chatbot selection, and they seldom described a prompt engineering phase. Authors infrequently shared prompt and chatbot response transcripts. Less than half mentioned ethical issues, patient safety, or regulation of LLMs.

### Reporting of LLM Characteristics and Study Method

Generative AI-driven chatbots constitute the intervention in these studies, yet there is a lack of critical information about their characteristics. As these publications are largely prepared by clinicians, study teams may not have access to expertise in machine learning. Without full knowledge of the intervention being applied, it is difficult to interpret LLM performance.^9,17,156^

LLMs are trained to learn associations between words in large training text datasets,^2^ transform algorithm inputs to outputs, and mimic human language using NLP.^3^ This process enables LLMs to predict the next word in a sequence, and preset model characteristics, such as temperature, token length, fine-tuning availability, penalties, add-on availability, language, and layers, further impact their functionality.^157,158,159^ Just as sufficient detail about medical or surgical interventions must be reported in traditional studies to reproduce experiments and guide critical appraisal, LLM characteristics are similarly vital. Physicians may not be AI experts, but they should understand the principles and limitations of their LLM interventions and may benefit from involving expert LLM researchers.^160^

Most included studies did not report a prompt engineering phase. However, the generated output depends greatly on the initial input. LLMs have in-context learning capabilities that allow them to adapt to the given prompt, so prompts can be intentionally designed to support performance on further tasks, such as patient communication, administrative tasks, risk assessments, or clinical decision-making.^160,161^ Prompt engineering improves chatbot performance by tailoring LLM output to be focused and helpful for a specific task.^162,163^ These crucial concepts underscore the importance of including experts in machine learning, computer science, and engineering. In the push for the clinical integration of LLMs in medicine, multidisciplinary stakeholder collaboration between physicians and expert LLM and NLP researchers is essential.^164^

Additionally, the existing literature base emphasizes a structured approach to performance evaluation.^165^ However, most studies did not provide transcripts of chatbot prompts and responses. Inclusion of obtained responses and the details of subsequent analysis is important to maximize transparency, internal validity, and the external appraisal of chatbot assessment study findings. Authors frequently apply subjective measures of chatbot performance, such as expert opinion, in CHAS, limiting the generalizability of their methods and conclusions. A shift toward objective measures, preferably basing accuracy and relevance with respect to high-quality evidence, such as clinical practice guidelines, is necessary. Many studies describe this as defining a ground truth or evaluation dataset, and it is an essential step in LLM performance evaluation.^166,167^ Currently, few studies use clinical practice guidelines to define successful performance, and several chatbots have experienced inconsistent accuracy by these standards.^168,169^ This is especially important when considering the implications of using these LLMs on patient safety.

### Ethics, Patient Safety, and LLM Regulation

Artificial intelligence–linked medical devices are considered software as a medical device and are regulated under federal departments such as Health Canada and the US Food and Drug Administration.^170^ However, LLM applications can be excluded from this regulation if they were not designed for medical purposes or intended to replace clinical judgment.^11,171,172^ Although no devices that use generative AI or LLMs have been approved by the Food and Drug Administration,^173^ LLMs are being used in medical applications through this gap in regulation.^11^ Some LLMs have already been integrated into electronic medical record systems,^174^ such as the Nuance DAX Copilot, a generative AI-program embedded in an electronic health record software called Epic, which records patient interactions and automatically produces clinical documentation.^175,176^ To safely implement generative AI-driven chatbots in practice, there is a need for updated regulation that addresses the differences between emerging generative AI and previous forms of AI.^11^ While other regulated AI medical technologies, such as radiology image analysis^177^ or diagnosis,^178^ are trained on domain-specific medical data for targeted uses, LLMs have broader complexity, applicability, and an ability to adapt in real time, which complicates their regulation.^2,179,180^

Additionally, the specific issues of algorithmic bias, hallucinations, and risks of privacy breaches must be addressed by regulatory standards,^181,182^ as these concepts have ethical implications in patient care.^183^ Several examples of bias have been reported among LLMs.^184^ These have included the propagation of biases based on race and ethnicity, gender, educational level, social class, sexuality, and age.^184,185,186^ Many of these biases stem from the data used to train LLMs.^184^ Additionally, LLMs have been known to produce hallucinations.^2,187^ Large language model performance is largely influenced by the content used for pretraining and fine-tuning, yet LLMs are generally not trained exclusively with high-quality medical literature or real-world clinical data.^188^ While clinical practice is informed by evidence from peer-reviewed research, LLMs replicate their input data and thus may propagate online health misinformation and prejudices,^189,190^ compromising patient safety. Moreover, the lack of transparency surrounding the development and external validation of closed-source or proprietary LLMs presents additional challenges to their evaluation for health care purposes.^179,180^ Concerns have also been raised related to data privacy of patient health information among proprietary LLMs.^182,191^ There is a potential for misuse of patient data, medicolegal implications, and loss of physician-patient trust.^181,192^

Taken together, these factors raise major issues as we move from the technological development of LLMs to their clinical validation for use in health care settings.^182^ The World Health Organization released guidance on ethics and governance of AI for health,^182^ which overlaps substantially with LLMs, which are unimodal models. This document outlines key messages for stakeholders involved in the health care integration of generative AI. For instance, the World Health Organization calls for developers to commit to ethical principles of inclusiveness, use higher-quality data during model development, adhere to data protection laws, be transparent about model training data, and involve key stakeholders in the design and development of LLMs, including health care professionals, patients, laypersons, and vulnerable persons.^182^ Moreover, developers may focus on the explainability of their LLMs, a concept that aims to elucidate how models arrive at outputs given a specific input.^193^ In addition, governments should develop clear regulatory policies and legislation to capture many of the concepts discussed herein, such as target product profiles, design and development standards, audits, disclosure requirements, data protection, and public infrastructure for the development of LLMs. Regulators may further look toward incentivizing researchers toward the development of standardized metrics for the clinical validation of health care applications of generative AI models such as LLMs.^193^

### Limitations

This systematic review has limitations. First, the reporting used in CHAS may change beyond the time of this review due to the dynamic nature of this field. By engineering a comprehensive search strategy, we aimed to capture an accurate representation of the literature. Second, much of the literature evaluated in this review used hypothetical patient cases. CHAS should focus on patient-centered, prospective research using LLMs in the clinical setting. Multidisciplinary collaboration between clinicians and LLM/NLP experts may help facilitate this, while also improving the understanding and reporting of LLM characteristics in these studies. In addition, there is a paucity of literature that comments in detail on the ethical and regulatory considerations of the clinical integration of LLMs that impact patient safety. Clinicians, patients, and regulators should be involved in collective efforts to agree and enforce appropriate deployment strategies for LLM technology in medicine.

Standardized frameworks are under development to establish detailed reporting standards to help authors design and report these studies.^18^ The CHART reporting tool may guide transparent reporting of CHAS and promote the publication of CHAS with higher methodologic rigor. Future research should address the lack of quality appraisal tools available to evaluate CHAS. Moreover, standardized metrics to validate the health care application of generative AI models are needed to facilitate their evaluation for adoption in clinical pathways. Interdisciplinary work among hospital managers, policymakers, regulatory bodies, and physicians of varying specialty backgrounds should be conducted to respond to the rapid advancement in LLM technology and the push for clinical integration by producing regulatory frameworks to support the safe clinical integration of LLMs.

## Conclusions

In this systematic review of 137 CHAS, we noted that detailed and transparent reporting of key aspects often was not included in the studies, such as LLM characteristics, prompt engineering, query strategy, and performance evaluation. An emphasis should be placed on high-quality methods to justify the deployment of novel applications of LLMs and related technologies in clinical practice.

## Notes

Supplement 1. **eAppendix.** Literature Search Syntax

Supplement 2.
Data Sharing Statement

## References

[1] Singhal K, Azizi S, Tu T, . Large language models encode clinical knowledge. Nature. 2023;620(7972):172-180. doi:10.1038/s41586-023-06291-2 37438534 PMC10396962

[2] Thirunavukarasu AJ, Ting DSJ, Elangovan K, Gutierrez L, Tan TF, Ting DSW. Large language models in medicine. Nat Med. 2023;29(8):1930-1940. doi:10.1038/s41591-023-02448-8 37460753

[3] Khurana D, Koli A, Khatter K, Singh S. Natural language processing: state of the art, current trends and challenges. Multimed Tools Appl. 2023;82(3):3713-3744. doi:10.1007/s11042-022-13428-4 35855771 PMC9281254

[4] Battineni G, Baldoni S, Chintalapudi N, . Factors affecting the quality and reliability of online health information. Digit Health. 2020;6:2055207620948996. doi:10.1177/2055207620948996 32944269 PMC7466903

[5] Shen SA, Perez-Heydrich CA, Xie DX, Nellis JC. ChatGPT vs. web search for patient questions: what does ChatGPT do better? Eur Arch Otorhinolaryngol. 2024;281(6):3219-3225. doi:10.1007/s00405-024-08524-0 38416195 PMC11410109

[6] Ayers JW, Poliak A, Dredze M, . Comparing physician and artificial intelligence chatbot responses to patient questions posted to a public social media forum. JAMA Intern Med. 2023;183(6):589-596. doi:10.1001/jamainternmed.2023.1838 37115527 PMC10148230

[7] Shahsavar Y, Choudhury A. User intentions to use ChatGPT for self-diagnosis and health-related purposes: cross-sectional survey study. JMIR Hum Factors. 2023;10:e47564. doi:10.2196/47564 37195756 PMC10233444

[8] Temsah MH, Altamimi I, Jamal A, Alhasan K, Al-Eyadhy A. ChatGPT surpasses 1000 publications on PubMed: envisioning the road ahead. Cureus. 2023;15(9):e44769. doi:10.7759/cureus.44769 37809155 PMC10557088

[9] Lee P, Bubeck S, Petro J. Benefits, limits, and risks of GPT-4 as an AI chatbot for medicine. N Engl J Med. 2023;388(13):1233-1239. doi:10.1056/NEJMsr2214184 36988602

[10] Li J, Dada A, Puladi B, Kleesiek J, Egger J. ChatGPT in healthcare: a taxonomy and systematic review. Comput Methods Programs Biomed. 2024;245:108013. doi:10.1016/j.cmpb.2024.108013 38262126

[11] Meskó B, Topol EJ. The imperative for regulatory oversight of large language models (or generative AI) in healthcare. NPJ Digit Med. 2023;6(1):120. doi:10.1038/s41746-023-00873-0 37414860 PMC10326069

[12] Sallam M. ChatGPT utility in healthcare education, research, and practice: systematic review on the promising perspectives and valid concerns. Healthcare (Basel). 2023;11(6):887. doi:10.3390/healthcare11060887 36981544 PMC10048148

[13] Davis R, Eppler M, Ayo-Ajibola O, . Evaluating the effectiveness of artificial intelligence–powered large language models application in disseminating appropriate and readable health information in urology. J Urol. 2023;210(4):688-694. doi:10.1097/JU.0000000000003615 37428117

[14] Gilson A, Safranek CW, Huang T, . How does ChatGPT perform on the United States Medical Licensing Examination (USMLE)? the implications of large language models for medical education and knowledge assessment. JMIR Med Educ. 2023;9:e45312. doi:10.2196/45312 36753318 PMC9947764

[15] Williams SC, Starup-Hansen J, Funnell JP, . Can ChatGPT outperform a neurosurgical trainee? a prospective comparative study. Br J Neurosurg. 2024:1-10. doi:10.1080/02688697.2024.2308222 38305239 PMC12090375

[16] Ye C, Zweck E, Ma Z, Smith J, Katz S. Doctor versus artificial intelligence: patient and physician evaluation of large language model responses to rheumatology patient questions in a cross-sectional study. Arthritis Rheumatol. 2024;76(3):479-484. doi:10.1002/art.42737 37902018

[17] Moher D, Schulz KF, Simera I, Altman DG. Guidance for developers of health research reporting guidelines. PLoS Med. 2010;7(2):e1000217. doi:10.1371/journal.pmed.1000217 20169112 PMC2821895

[18] Huo B, Cacciamani GE, Collins GS, McKechnie T, Lee Y, Guyatt G. Reporting standards for the use of large language model-linked chatbots for health advice. Nat Med. 2023;29(12):2988. doi:10.1038/s41591-023-02656-2 37957381

[19] Page MJ, McKenzie JE, Bossuyt PM, . The PRISMA 2020 statement: an updated guideline for reporting systematic reviews. BMJ. 2021;372(71):n71. doi:10.1136/bmj.n71 33782057 PMC8005924

[20] Registries OSF. Protocol for a scoping review of chatbot assessment studies: guidance for the CHART tool. February 25, 2024. Accessed December 9, 2024. https://osf.io/cxsk3

[21] Al-Ashwal FY, Zawiah M, Gharaibeh L, Abu-Farha R, Bitar AN. Evaluating the sensitivity, specificity, and accuracy of ChatGPT-3.5, ChatGPT-4, Bing AI, and Bard against conventional drug-drug interactions clinical tools. Drug Healthc Patient Saf. 2023;15:137-147. doi:10.2147/DHPS.S425858 37750052 PMC10518176

[22] Alessandri-Bonetti M, Giorgino R, Naegeli M, Liu HY, Egro FM. Assessing the soft tissue infection expertise of ChatGPT and Bard compared to IDSA recommendations. Ann Biomed Eng. 2024;52(6):1551-1553. doi:10.1007/s10439-023-03372-1 37865615

[23] Ali MJ. ChatGPT and lacrimal drainage disorders: performance and scope of improvement. Ophthalmic Plast Reconstr Surg. 2023;39(3):221-225. doi:10.1097/IOP.0000000000002418 37166289 PMC10171282

[24] Ali H, Patel P, Obaitan I, . Evaluating the performance of ChatGPT in responding to questions about endoscopic procedures for patients. iGIE. 2023;2(4):553-559. https://www.igiejournal.org/article/S2949-7086(23)00120-6/fulltext 10.1016/j.igie.2023.10.001 PMC12850862 41646058

[25] Altamimi I, Altamimi A, Alhumimidi AS, Altamimi A, Temsah MH. Snakebite advice and counseling from artificial intelligence: an acute venomous snakebite consultation with ChatGPT. Cureus. 2023;15(6):e40351. doi:10.7759/cureus.40351 37456381 PMC10339276

[26] Athavale A, Baier J, Ross E, Fukaya E. The potential of chatbots in chronic venous disease patient management. JVS Vasc Insights. 2023;1:100019. doi:10.1016/j.jvsvi.2023.100019 37701430 PMC10497234

[27] Ayers JW, Zhu Z, Poliak A, . Evaluating artificial intelligence responses to public health questions. JAMA Netw Open. 2023;6(6):e2317517. doi:10.1001/jamanetworkopen.2023.17517 37285160 PMC10248742

[28] Ayoub M, Ballout AA, Zayek RA, Ayoub NF. Mind + machine: ChatGPT as a basic clinical decisions support tool. Cureus. 2023;15(8):e43690. doi:10.7759/cureus.43690 37724211 PMC10505276

[29] Ayoub NF, Lee YJ, Grimm D, Balakrishnan K. Comparison between ChatGPT and Google search as sources of postoperative patient instructions. JAMA Otolaryngol Head Neck Surg. 2023;149(6):556-558. doi:10.1001/jamaoto.2023.0704 37103921 PMC10141286

[30] Balel Y. Can ChatGPT be used in oral and maxillofacial surgery? J Stomatol Oral Maxillofac Surg. 2023;124(5):101471. doi:10.1016/j.jormas.2023.101471 37061037

[31] Bellinger JR, De La Chapa JS, Kwak MW, Ramos GA, Morrison D, Kesser BW. BPPV information on Google versus AI (ChatGPT). Otolaryngol Head Neck Surg. 2024;170(6):1504-1511. doi:10.1002/ohn.506 37622581

[32] Benirschke RC, Wodskow J, Prasai K, Freeman A, Lee JM, Groth J. Assessment of a large language model’s utility in helping pathology professionals answer general knowledge pathology questions. Am J Clin Pathol. 2024;161(1):42-48. doi:10.1093/ajcp/aqad106 37658808

[33] Bernstein IA, Zhang YV, Govil D, . Comparison of ophthalmologist and large language model chatbot responses to online patient eye care questions. JAMA Netw Open. 2023;6(8):e2330320. doi:10.1001/jamanetworkopen.2023.30320 37606922 PMC10445188

[34] Biswas S, Logan NS, Davies LN, Sheppard AL, Wolffsohn JS. Assessing the utility of ChatGPT as an artificial intelligence-based large language model for information to answer questions on myopia. Ophthalmic Physiol Opt. 2023;43(6):1562-1570. doi:10.1111/opo.13207 37476960

[35] Caglar U, Yildiz O, Meric A, . Evaluating the performance of ChatGPT in answering questions related to pediatric urology. J Pediatr Urol. 2024;20(1):26.e1-26.e5. doi:10.1016/j.jpurol.2023.08.003 37596194

[36] Cakir H, Caglar U, Yildiz O, Meric A, Ayranci A, Ozgor F. Evaluating the performance of ChatGPT in answering questions related to urolithiasis. Int Urol Nephrol. 2024;56(1):17-21. doi:10.1007/s11255-023-03773-0 37658948

[37] Campbell DJ, Estephan LE, Mastrolonardo EV, Amin DR, Huntley CT, Boon MS. Evaluating ChatGPT responses on obstructive sleep apnea for patient education. J Clin Sleep Med. 2023;19(12):1989-1995. doi:10.5664/jcsm.10728 37485676 PMC10692937

[38] Caruccio L, Cirillo S, Polese G, Solimando G, Sundaramurthy S, Tortora G. Can ChatGPT provide intelligent diagnoses? a comparative study between predictive models and ChatGPT to define a new medical diagnostic bot. Expert Syst Appl. 2024;235(7):121186. doi:10.1016/j.eswa.2023.121186

[39] Chee J, Kwa ED, Goh X. “Vertigo, likely peripheral”: the dizzying rise of ChatGPT. Eur Arch Otorhinolaryngol. 2023;280(10):4687-4689. doi:10.1007/s00405-023-08135-1 37493845

[40] Chen S, Kann BH, Foote MB, . Use of artificial intelligence chatbots for cancer treatment information. JAMA Oncol. 2023;9(10):1459-1462. doi:10.1001/jamaoncol.2023.2954 37615976 PMC10450584

[41] Chervenak J, Lieman H, Blanco-Breindel M, Jindal S. The promise and peril of using a large language model to obtain clinical information: ChatGPT performs strongly as a fertility counseling tool with limitations. Fertil Steril. 2023;120(3, pt 2):575-583. doi:10.1016/j.fertnstert.2023.05.151 37217092

[42] Chiesa-Estomba CM, Lechien JR, Vaira LA, . Exploring the potential of Chat-GPT as a supportive tool for sialendoscopy clinical decision making and patient information support. Eur Arch Otorhinolaryngol. 2024;281(4):2081-2086. doi:10.1007/s00405-023-08104-8 37405455

[43] Chowdhury M, Lim E, Higham A, . can large language models safely address patient questions following cataract surgery? Invest Ophthalmol Vis Sci. 2023;64(8):1214. doi:10.18653/v1/2023.clinicalnlp-1.17

[44] Cocci A, Pezzoli M, Lo Re M, . Quality of information and appropriateness of ChatGPT outputs for urology patients. Prostate Cancer Prostatic Dis. 2024;27(1):103-108. doi:10.1038/s41391-023-00705-y 37516804

[45] Coskun BN, Yagiz B, Ocakoglu G, Dalkilic E, Pehlivan Y. Assessing the accuracy and completeness of artificial intelligence language models in providing information on methotrexate use. Rheumatol Int. 2024;44(3):509-515. doi:10.1007/s00296-023-05473-5 37747564

[46] Coskun B, Ocakoglu G, Yetemen M, Kaygisiz O. Can ChatGPT, an artificial intelligence language model, provide accurate and high-quality patient information on prostate cancer? Urology. 2023;180:35-58. doi:10.1016/j.urology.2023.05.040 37406864

[47] Cox A, Seth I, Xie Y, Hunter-Smith DJ, Rozen WM. Utilizing ChatGPT-4 for providing medical information on blepharoplasties to patients. Aesthet Surg J. 2023;43(8):NP658-NP662. doi:10.1093/asj/sjad096 37032521

[48] Deiana G, Dettori M, Arghittu A, Azara A, Gabutti G, Castiglia P. Artificial intelligence and public health: evaluating ChatGPT responses to vaccination myths and misconceptions. Vaccines (Basel). 2023;11(7):1217. doi:10.3390/vaccines11071217 37515033 PMC10386180

[49] Dubin JA, Bains SS, Chen Z, . Using a Google web search analysis to assess the utility of ChatGPT in total joint arthroplasty. J Arthroplasty. 2023;38(7):1195-1202. doi:10.1016/j.arth.2023.04.007 37040823

[50] Dwyer T, Hoit G, Burns D, . Use of an artificial intelligence conversational agent (chatbot) for hip arthroscopy patients following surgery. Arthrosc Sports Med Rehabil. 2023;5(2):e495-e505. doi:10.1016/j.asmr.2023.01.020 37101866 PMC10123501

[51] Emile SH, Horesh N, Freund M, . How appropriate are answers of online chat-based artificial intelligence (ChatGPT) to common questions on colon cancer? Surgery. 2023;174(5):1273-1275. doi:10.1016/j.surg.2023.06.005 37482439

[52] Endo Y, Sasaki K, Moazzam Z, . Quality of ChatGPT responses to questions related to liver transplantation. J Gastrointest Surg. 2023;27(8):1716-1719. doi:10.1007/s11605-023-05714-9 37254022

[53] Farhat F. ChatGPT as a complementary mental health resource: a boon or a bane. Ann Biomed Eng. 2024;52(5):1111-1114. doi:10.1007/s10439-023-03326-7 37477707

[54] Franco D’Souza R, Amanullah S, Mathew M, Surapaneni KM. Appraising the performance of ChatGPT in psychiatry using 100 clinical case vignettes. Asian J Psychiatr. 2023;89:103770. doi:10.1016/j.ajp.2023.103770 37812998

[55] Fraser H, Crossland D, Bacher I, Ranney M, Madsen T, Hilliard R. Comparison of diagnostic and triage accuracy of ada health and WebMD symptom checkers, ChatGPT, and physicians for patients in an emergency department: clinical data analysis study. JMIR Mhealth Uhealth. 2023;11(1):e49995. doi:10.2196/49995 37788063 PMC10582809

[56] Gabriel J, Shafik L, Alanbuki A, Larner T. The utility of the ChatGPT artificial intelligence tool for patient education and enquiry in robotic radical prostatectomy. Int Urol Nephrol. 2023;55(11):2717-2732. doi:10.1007/s11255-023-03729-4 37528247

[57] Galido PV, Butala S, Chakerian M, Agustines D. A case study demonstrating applications of ChatGPT in the clinical management of treatment-resistant schizophrenia. Cureus. 2023;15(4):e38166. doi:10.7759/cureus.38166 37252576 PMC10219639

[58] Gebrael G, Sahu KK, Chigarira B, . Enhancing triage efficiency and accuracy in emergency rooms for patients with metastatic prostate cancer: a retrospective analysis of artificial intelligence-assisted triage using ChatGPT 4.0. Cancers (Basel). 2023;15(14):3717. doi:10.3390/cancers15143717 37509379 PMC10378202

[59] Goodman RS, Patrinely JR, Stone CA Jr, . Accuracy and reliability of chatbot responses to physician questions. JAMA Netw Open. 2023;6(10):e2336483. doi:10.1001/jamanetworkopen.2023.36483 37782499 PMC10546234

[60] Gordon EB, Towbin AJ, Wingrove P, . Enhancing patient communication with Chat-GPT in radiology: evaluating the efficacy and readability of answers to common imaging-related questions. J Am Coll Radiol. 2024;21(2):353-359. doi:10.1016/j.jacr.2023.09.011 37863153

[61] Gracias D, Siu A, Seth I, Dooreemeah D, Lee A. Exploring the role of an artificial intelligence chatbot on appendicitis management: an experimental study on ChatGPT. ANZ J Surg. 2024;94(3):342-352. doi:10.1111/ans.18736 37855397

[62] Gravel J, D’Amours-Gravel M, Osmanlliu E. Learning to fake it: limited responses and fabricated references provided by ChatGPT for medical questions. Mayo Clin Proc Digit Health. 2023;1(3):226-234. doi:10.1016/j.mcpdig.2023.05.004 PMC11975740 40206627

[63] Gwak G, Hwang U, Jung S, Kim J. Search for medical information and treatment options for musculoskeletal disorders through an artificial intelligence chatbot: focusing on shoulder impingement syndrome. J Musculoskelet Sci Technol. 2023;7(1):8-16. doi:10.29273/jmst.2023.7.1.8

[64] Haemmerli J, Sveikata L, Nouri A, . ChatGPT in glioma adjuvant therapy decision making: ready to assume the role of a doctor in the tumour board? BMJ Health Care Inform. 2023;30(1):e100775. doi:10.1136/bmjhci-2023-100775 37399360 PMC10314415

[65] Harskamp RE, De Clercq L. Performance of ChatGPT as an AI-assisted decision support tool in medicine: a proof-of-concept study for interpreting symptoms and management of common cardiac conditions (AMSTELHEART-2). Acta Cardiol. 2024;79(3):358-366. doi:10.1080/00015385.2024.2303528 38348835

[66] Haver HL, Lin CT, Sirajuddin A, Yi PH, Jeudy J. Evaluating ChatGPT’s accuracy in lung cancer prevention and screening recommendations. Radiol Cardiothorac Imaging. 2023;5(4):e230115. doi:10.1148/ryct.230115 37693201 PMC10483248

[67] Haver HL, Ambinder EB, Bahl M, Oluyemi ET, Jeudy J, Yi PH. Appropriateness of breast cancer prevention and screening recommendations provided by ChatGPT. Radiology. 2023;307(4):e230424. doi:10.1148/radiol.230424 37014239

[68] Hirosawa T, Harada Y, Yokose M, Sakamoto T, Kawamura R, Shimizu T. Diagnostic accuracy of differential-diagnosis lists generated by generative pretrained transformer 3 chatbot for clinical vignettes with common chief complaints: a pilot study. Int J Environ Res Public Health. 2023;20(4):3378. doi:10.3390/ijerph20043378 36834073 PMC9967747

[69] Hirosawa T, Kawamura R, Harada Y, . ChatGPT-generated differential diagnosis lists for complex case-derived clinical vignettes: diagnostic accuracy evaluation. JMIR Med Inform. 2023;11:e48808. doi:10.2196/48808 37812468 PMC10594139

[70] Hirosawa T, Mizuta K, Harada Y, Shimizu T. Comparative evaluation of diagnostic accuracy between google bard and physicians. Am J Med. 2023;136(11):1119-1123.e18. doi:10.1016/j.amjmed.2023.08.003 37643659

[71] Hopkins AM, Logan JM, Kichenadasse G, Sorich MJ. Artificial intelligence chatbots will revolutionize how cancer patients access information: ChatGPT represents a paradigm-shift. J Natl Cancer Inst Cancer Spectr. 2023;7(2):pkad010. doi:10.1093/jncics/pkad010 36808255 PMC10013638

[72] Hristidis V, Ruggiano N, Brown EL, Ganta SRR, Stewart S. ChatGPT vs Google for queries related to dementia and other cognitive decline: comparison of results. J Med Internet Res. 2023;25:e48966. doi:10.2196/48966 37490317 PMC10410383

[73] Hurley ET, Crook BS, Lorentz SG, . Evaluation high-quality of information from ChatGPT (artificial intelligence-large language model) artificial intelligence on shoulder stabilization surgery. Arthroscopy. 2024;40(3):726-731.e6. doi:10.1016/j.arthro.2023.07.048 37567487

[74] Janopaul-Naylor JR, Koo A, Qian DC, McCall NS, Liu Y, Patel SA. Physician assessment of ChatGPT and Bing answers to American Cancer Society’s questions to Ask About Your Cancer. Am J Clin Oncol. 2024;47(1):17-21. doi:10.1097/COC.0000000000001050 37823708 PMC10841271

[75] Johnson D, Goodman R, Patrinely J, . Assessing the accuracy and reliability of AI-generated medical responses: an evaluation of the Chat-GPT model. Res Sq. Preprint posted online February 28, 2023. doi:10.21203/rs.3.rs-2566942/v1

[76] Kao HJ, Chien TW, Wang WC, Chou W, Chow JC. Assessing ChatGPT’s capacity for clinical decision support in pediatrics: a comparative study with pediatricians using KIDMAP of Rasch analysis. Medicine (Baltimore). 2023;102(25):e34068. doi:10.1097/MD.0000000000034068 37352054 PMC10289633

[77] Kataoka Y, Takemura T, Sasajima M, Katoh N. Development and early feasibility of chatbots for educating patients with lung cancer and their caregivers in Japan: Mixed methods study. JMIR Cancer. 2021;7(1):e26911. doi:10.2196/26911 33688839 PMC8086641

[78] King RC, Samaan JS, Yeo YH, Mody B, Lombardo DM, Ghashghaei R. Appropriateness of ChatGPT in answering heart failure related questions. Heart Lung Circ. 2024;33(9):1314-1318. doi:10.1016/j.hlc.2024.03.005 38821760

[79] Koh SJQ, Yeo KK, Yap JJL. Leveraging ChatGPT to aid patient education on coronary angiogram. Ann Acad Med Singap. 2023;52(7):374-377. doi:10.47102/annals-acadmedsg.2023138 38904503

[80] Krusche M, Callhoff J, Knitza J, Ruffer N. Diagnostic accuracy of a large language model in rheumatology: comparison of physician and ChatGPT-4. Rheumatol Int. 2024;44(2):303-306. doi:10.1007/s00296-023-05464-6 37742280 PMC10796566

[81] Kuroiwa T, Sarcon A, Ibara T, . The potential of ChatGPT as a self-diagnostic tool in common orthopedic diseases: exploratory study. J Med Internet Res. 2023;25(1):e47621. doi:10.2196/47621 37713254 PMC10541638

[82] Kusunose K, Kashima S, Sata M. Evaluation of the accuracy of ChatGPT in answering clinical questions on the Japanese Society of Hypertension guidelines. Circ J. 2023;87(7):1030-1033. doi:10.1253/circj.CJ-23-0308 37286486

[83] Lahat A, Shachar E, Avidan B, Glicksberg B, Klang E. Evaluating the utility of a large language model in answering common patients’ gastrointestinal health–related questions: are we there yet? Diagnostics (Basel). 2023;13(11):1950. doi:10.3390/diagnostics13111950 37296802 PMC10252924

[84] Lam CS, Hua R, Koon HK, . Can ChatGPT provide quality information on integrative oncology? a brief report. J Integr Complement Med. 2024;30(2):196-205. doi:10.1089/jicm.2023.0290 37792344

[85] Lechien JR, Georgescu BM, Hans S, Chiesa-Estomba CM. ChatGPT performance in laryngology and head and neck surgery: a clinical case-series. Eur Arch Otorhinolaryngol. 2024;281(1):319-333. doi:10.1007/s00405-023-08282-5 37874336

[86] Lee TC, Staller K, Botoman V, Pathipati MP, Varma S, Kuo B. ChatGPT answers common patient questions about colonoscopy. Gastroenterology. 2023;165(2):509-511.e7. doi:10.1053/j.gastro.2023.04.033 37150470

[87] Levartovsky A, Ben-Horin S, Kopylov U, Klang E, Barash Y. Towards AI-augmented clinical decision-making: an examination of ChatGPT’s utility in acute ulcerative colitis presentations. Am J Gastroenterol. 2023;118(12):2283-2289. doi:10.14309/ajg.0000000000002483 37611254

[88] Levkovich I, Elyoseph Z. Identifying depression and its determinants upon initiating treatment: ChatGPT versus primary care physicians. Fam Med Community Health. 2023;11(4):e002391. doi:10.1136/fmch-2023-002391 37844967 PMC10582915

[89] Levkovich I, Elyoseph Z. suicide risk assessments through the eyes of ChatGPT-3.5 versus ChatGPT-4: vignette study. JMIR Ment Health. 2023;10(1):e51232. doi:10.2196/51232 37728984 PMC10551796

[90] Li J, Gao X, Dou T, Gao Y, Zhu W. Assessing the performance of GPT-4 in the filed of osteoarthritis and orthopaedic case consultation. Published online August 9, 2023. MedRxiv.

[91] Li W, Chen J, Chen F, Liang J, Yu H. Exploring the potential of ChatGPT-4 in responding to common questions about abdominoplasty: an AI-based case study of a plastic surgery consultation. Aesthetic Plast Surg. 2024;48(8):1571-1583. doi:10.1007/s00266-023-03660-0 37770637

[92] Lim ZW, Pushpanathan K, Yew SME, . Benchmarking large language models’ performances for myopia care: a comparative analysis of ChatGPT-3.5, ChatGPT-4.0, and Google Bard. EBioMedicine. 2023;95:104770. doi:10.1016/j.ebiom.2023.104770 37625267 PMC10470220

[93] Lim DYZ, Tan YB, Koh JTE, . ChatGPT on guidelines: providing contextual knowledge to GPT allows it to provide advice on appropriate colonoscopy intervals. J Gastroenterol Hepatol. 2024;39(1):81-106. doi:10.1111/jgh.16375 37855067

[94] Liu J, Zheng J, Cai X, Wu D, Yin C. A descriptive study based on the comparison of ChatGPT and evidence-based neurosurgeons. iScience. 2023;26(9):107590. doi:10.1016/j.isci.2023.107590 37705958 PMC10495632

[95] Liu HY, Alessandri Bonetti M, Jeong T, Pandya S, Nguyen VT, Egro FM. Dr. ChatGPT will see you now: how do Google and ChatGPT compare in answering patient questions on breast reconstruction? J Plast Reconstr Aesthet Surg. 2023;85:488-497. doi:10.1016/j.bjps.2023.07.039 37598590

[96] Liu S, Wright AP, Paterson BL, . Assessing the value of ChatGPT for clinical decision support optimization. Peprint posted online February 23, 2023. MedRxiv.

[97] Long C, Subburam D, Lowe K, . ChatENT: augmented large language model for expert knowledge retrieval in otolaryngology-head and neck surgery. Otolaryngol Head Neck Surg. 2024;171(4):1042-1051. doi:10.1002/ohn.864 38895862

[98] Lower K, Seth I, Lim B, Seth N. ChatGPT-4: transforming medical education and addressing clinical exposure challenges in the post-pandemic era. Indian J Orthop. 2023;57(9):1527-1544. doi:10.1007/s43465-023-00967-7 37609022 PMC10442004

[99] Luykx JJ, Gerritse F, Habets PC, Vinkers CH. The performance of ChatGPT in generating answers to clinical questions in psychiatry: a two-layer assessment. World Psychiatry. 2023;22(3):479-480. doi:10.1002/wps.21145 37713576 PMC10503909

[100] Lyons RJ, Arepalli SR, Fromal O, Choi JD, Jain N. Artificial intelligence chatbot performance in triage of ophthalmic conditions. Can J Ophthalmol. 2024;59(4):e301-e308. doi:10.1016/j.jcjo.2023.07.016 37572695

[101] Maillard A, Micheli G, Lefevre L, . Can chatbot artificial intelligence replace infectious diseases physicians in the management of bloodstream infections? a prospective cohort study. Clin Infect Dis. 2024;78(4):825-832. doi:10.1093/cid/ciad632 37823416

[102] Manolitsis I, Feretzakis G, Tzelves L, . Training ChatGPT models in assisting urologists in daily practice. Stud Health Technol Inform. 2023;305:576-579. doi:10.3233/SHTI230562 37387096

[103] Mehnen L, Gruarin S, Vasileva M, Knapp B. ChatGPT as a medical doctor? a diagnostic accuracy study on common and rare diseases. Preprint posted online April 26, 2023. MedRxiv.

[104] Mesnier J, Suc G, Sayah N, Abtan J, Steg PG. Relevance of medical information obtained from ChatGPT: are large language models friends or foes? Arch Cardiovasc Dis. 2023;116(10):485-486. doi:10.1016/j.acvd.2023.07.009 37718185

[105] Mika AP, Martin JR, Engstrom SM, Polkowski GG, Wilson JM. Assessing ChatGPT responses to common patient questions regarding total hip arthroplasty. J Bone Joint Surg Am. 2023;105(19):1519-1526. doi:10.2106/JBJS.23.00209 37459402

[106] Mishra A, Begley SL, Chen A, . Exploring the intersection of artificial intelligence and neurosurgery: let us be cautious with ChatGPT. Neurosurgery. 2023;93(6):1366-1373. doi:10.1227/neu.0000000000002598 37417886

[107] Momenaei B, Wakabayashi T, Shahlaee A, . Appropriateness and readability of ChatGPT-4–generated responses for surgical treatment of retinal diseases. Ophthalmol Retina. 2023;7(10):862-868. doi:10.1016/j.oret.2023.05.022 37277096

[108] Moshirfar M, Altaf AW, Stoakes IM, Tuttle JJ, Hoopes PC. Artificial intelligence in ophthalmology: a comparative analysis of GPT-3.5, GPT-4, and human expertise in answering StatPearls questions. Cureus. 2023;15(6):e40822. doi:10.7759/cureus.40822 37485215 PMC10362981

[109] Musheyev D, Pan A, Loeb S, Kabarriti AE. How well do artificial intelligence chatbots respond to the top search queries about urological malignancies? Eur Urol. 2024;85(1):13-16. doi:10.1016/j.eururo.2023.07.004 37567827

[110] Nastasi AJ, Courtright KR, Halpern SD, Weissman GE. A vignette-based evaluation of ChatGPT’s ability to provide appropriate and equitable medical advice across care contexts. Sci Rep. 2023;13(1):17885. doi:10.1038/s41598-023-45223-y 37857839 PMC10587094

[111] Nielsen JPS, von Buchwald C, Grønhøj C. Validity of the large language model ChatGPT (GPT4) as a patient information source in otolaryngology by a variety of doctors in a tertiary otorhinolaryngology department. Acta Otolaryngol. 2023;143(9):779-782. doi:10.1080/00016489.2023.2254809 37694729

[112] O’Hagan R, Kim RH, Abittan BJ, Caldas S, Ungar J, Ungar B. Trends in accuracy and appropriateness of alopecia areata information obtained from a popular online large language model, ChatGPT. Dermatology. 2023;239(6):952-957. doi:10.1159/000534005 37722370

[113] Padovan M, Cosci B, Petillo A, . ChatGPT in occupational medicine: a comparative study with human experts. Bioengineering (Basel). 2024;11(1):57. doi:10.3390/bioengineering11010057 38247934 PMC10813435

[114] Pan A, Musheyev D, Bockelman D, Loeb S, Kabarriti AE. Assessment of artificial intelligence chatbot responses to top searched queries about cancer. JAMA Oncol. 2023;9(10):1437-1440. doi:10.1001/jamaoncol.2023.2947 37615960 PMC10450581

[115] Potapenko I, Boberg-Ans LC, Stormly Hansen M, Klefter ON, van Dijk EHC, Subhi Y. Artificial intelligence–based chatbot patient information on common retinal diseases using ChatGPT. Acta Ophthalmol. 2023;101(7):829-831. doi:10.1111/aos.15661 36912780

[116] Potapenko I, Malmqvist L, Subhi Y, Hamann S. Artificial intelligence-based ChatGPT responses for patient questions on optic disc drusen. Ophthalmol Ther. 2023;12(6):3109-3119. doi:10.1007/s40123-023-00800-2 37698823 PMC10640407

[117] Qu RW, Qureshi U, Petersen G, Lee SC. Diagnostic and management applications of ChatGPT in structured otolaryngology clinical scenarios. OTO Open. 2023;7(3):e67. doi:10.1002/oto2.67 37614494 PMC10442607

[118] Rahsepar AA, Tavakoli N, Kim GHJ, Hassani C, Abtin F, Bedayat A. How AI responds to common lung cancer questions: ChatGPT vs Google Bard. Radiology. 2023;307(5):e230922. doi:10.1148/radiol.230922 37310252

[119] Rao A, Pang M, Kim J, . Assessing the utility of ChatGPT throughout the entire clinical workflow: development and usability study. J Med Internet Res. 2023;25:e48659. doi:10.2196/48659 37606976 PMC10481210

[120] Rao A, Kim J, Kamineni M, . Evaluating GPT as an adjunct for radiologic decision making: GPT-4 versus GPT-3.5 in a breast imaging pilot. J Am Coll Radiol. 2023;20(10):990-997. doi:10.1016/j.jacr.2023.05.003 37356806 PMC10733745

[121] Rasmussen MLR, Larsen AC, Subhi Y, Potapenko I. Artificial intelligence–based ChatGPT chatbot responses for patient and parent questions on vernal keratoconjunctivitis. Graefes Arch Clin Exp Ophthalmol. 2023;261(10):3041-3043. doi:10.1007/s00417-023-06078-1 37129631

[122] Rau A, Rau S, Zoeller D, . A context-based chatbot surpasses trained radiologists and generic ChatGPT in following the ACR appropriateness guidelines. Radiology. 2023;308(1):e230970. doi:10.1148/radiol.230970 37489981

[123] Rizwan A, Sadiq T. The use of AI in diagnosing diseases and providing management plans: a consultation on cardiovascular disorders with ChatGPT. Cureus. 2023;15(8):e43106. doi:10.7759/cureus.43106 37692649 PMC10483170

[124] Rogasch JMM, Metzger G, Preisler M, . ChatGPT: can you prepare my patients for [18F]FDG PET/CT and explain my reports? J Nucl Med. 2023;64(12):1876-1879. doi:10.2967/jnumed.123.266114 37709536 PMC10690125

[125] Rojas-Carabali W, Cifuentes-González C, Wei X, . Evaluating the diagnostic accuracy and management recommendations of ChatGPT in uveitis. Ocul Immunol Inflamm. 2024;32(8):1526-1531. doi:10.1080/09273948.2023.2253471 37722842

[126] Rosen S, Saban M. Can ChatGPT assist with the initial triage? a case study of stroke in young females. Int Emerg Nurs. 2023;70:101340. doi:10.1016/j.ienj.2023.101340

[127] Rosen S, Saban M. Evaluating the reliability of ChatGPT as a tool for imaging test referral: a comparative study with a clinical decision support system. Eur Radiol. 2024;34(5):2826-2837. doi:10.1007/s00330-023-10230-0 37828297

[128] Samaan JS, Yeo YH, Ng WH, . ChatGPT’s ability to comprehend and answer cirrhosis related questions in Arabic. Arab J Gastroenterol. 2023;24(3):145-148. doi:10.1016/j.ajg.2023.08.001 37673708

[129] Samaan JS, Yeo YH, Rajeev N, . Assessing the accuracy of responses by the language model ChatGPT to questions regarding bariatric surgery. Obes Surg. 2023;33(6):1790-1796. doi:10.1007/s11695-023-06603-5 37106269 PMC10234918

[130] Schulte B. Capacity of ChatGPT to identify guideline-based treatments for advanced solid tumors. Cureus. 2023;15(4):e37938. doi:10.7759/cureus.37938 37220429 PMC10200252

[131] Seth I, Cox A, Xie Y, . Evaluating chatbot efficacy for answering frequently asked questions in plastic surgery: a ChatGPT case study focused on breast augmentation. Aesthet Surg J. 2023;43(10):1126-1135. doi:10.1093/asj/sjad140 37158147

[132] Seth I, Lim B, Xie Y, . Comparing the efficacy of large language models ChatGPT, BARD, and Bing AI in providing information on rhinoplasty: an observational study. Aesthet Surg J Open Forum. 2023;5:ojad084. doi:10.1093/asjof/ojad084 37795257 PMC10547367

[133] Seth I, Xie Y, Rodwell A, . Exploring the role of a large language model on carpal tunnel syndrome management: an observation study of ChatGPT. J Hand Surg Am. 2023;48(10):1025-1033. doi:10.1016/j.jhsa.2023.07.003 37530687

[134] Sezgin E, Chekeni F, Lee J, Keim S. Clinical accuracy of large language models and Google search responses to postpartum depression questions: cross-sectional study. J Med Internet Res. 2023;25(1):e49240. doi:10.2196/49240 37695668 PMC10520763

[135] Shao CY, Li H, Liu XL, . Appropriateness and comprehensiveness of Using ChatGPT for perioperative patient education in thoracic surgery in different language contexts: survey study. Interact J Med Res. 2023;12:e46900. doi:10.2196/46900 37578819 PMC10463083

[136] Sorin V, Klang E, Sklair-Levy M, . Large language model (ChatGPT) as a support tool for breast tumor board. NPJ Breast Cancer. 2023;9(1):44. doi:10.1038/s41523-023-00557-8 37253791 PMC10229606

[137] Stevenson E, Walsh C, Hibberd L. Can artificial intelligence replace biochemists? a study comparing interpretation of thyroid function test results by ChatGPT and Google Bard to practising biochemists. Ann Clin Biochem. 2024;61(2):143-149. doi:10.1177/00045632231203473 37699796

[138] Stroop A, Stroop T, Zawy Alsofy S, . Large language models: are artificial intelligence–based chatbots a reliable source of patient information for spinal surgery? Eur Spine J. 2024;33(11):4135-4143. doi:10.1007/s00586-023-07975-z 37821602

[139] Suresh K, Rathi V, Nwosu O, . Utility of GPT-4 as an informational patient resource in otolaryngology. Published online May 16, 2023. MedRxiv.

[140] Szczesniewski JJ, Tellez Fouz C, Ramos Alba A, Diaz Goizueta FJ, García Tello A, Llanes González L. ChatGPT and most frequent urological diseases: analysing the quality of information and potential risks for patients. World J Urol. 2023;41(11):3149-3153. doi:10.1007/s00345-023-04563-0 37632558

[141] Vaira LA, Lechien JR, Abbate V, . Accuracy of ChatGPT-generated information on head and neck and oromaxillofacial surgery: a multicenter collaborative analysis. Otolaryngol Head Neck Surg. 2024;170(6):1492-1503. doi:10.1002/ohn.489 37595113

[142] Van Bulck L, Moons P. What if your patient switches from Dr. Google to Dr. ChatGPT? a vignette-based survey of the trustworthiness, value, and danger of ChatGPT-generated responses to health questions. Eur J Cardiovasc Nurs. 2024;23(1):95-98. doi:10.1093/eurjcn/zvad038 37094282

[143] Wagner MW, Ertl-Wagner BB. Accuracy of information and references using ChatGPT-3 for retrieval of clinical radiological information. Can Assoc Radiol J. 2024;75(1):69-73. doi:10.1177/08465371231171125 37078489

[144] Walker HL, Ghani S, Kuemmerli C, . Reliability of medical information provided by ChatGPT: assessment against clinical guidelines and patient information quality instrument. J Med Internet Res. 2023;25:e47479. doi:10.2196/47479 37389908 PMC10365578

[145] Wang Y, Visweswaran S, Kappor S, Kooragayalu S, Wu X. ChatGPT, enhanced with clinical practice guidelines, is a superior decision support tool. [published online August 13, 2023]. MedRxiv.

[146] Wang G, Liu Q, Chen G, . AI’s deep dive into complex pediatric inguinal hernia issues: a challenge to traditional guidelines? Hernia. 2023;27(6):1587-1599. doi:10.1007/s10029-023-02900-1 37843604

[147] Xie Y, Seth I, Hunter-Smith DJ, Rozen WM, Ross R, Lee M. Aesthetic surgery advice and counseling from artificial intelligence: a rhinoplasty consultation with ChatGPT. Aesthetic Plast Surg. 2023;47(5):1985-1993. doi:10.1007/s00266-023-03338-7 37095384 PMC10581928

[148] Yeo YH, Samaan JS, Ng WH, . Assessing the performance of ChatGPT in answering questions regarding cirrhosis and hepatocellular carcinoma. Clin Mol Hepatol. 2023;29(3):721-732. doi:10.3350/cmh.2023.0089 36946005 PMC10366809

[149] Yıldız MS, Alper A. Can ChatGPT-4 diagnose in Turkish: a comparison of ChatGPT responses to health-related questions in English and Turkish. J Consum Health Internet. 2023;27(3):294-307. doi:10.1080/15398285.2023.2240652

[150] Yun JY, Kim DJ, Lee N, Kim EK. A comprehensive evaluation of ChatGPT consultation quality for augmentation mammoplasty: a comparative analysis between plastic surgeons and laypersons. Int J Med Inform. 2023;179:105219. doi:10.1016/j.ijmedinf.2023.105219 37776670

[151] Zhou Z, Wang X, Li X, Liao L. Is ChatGPT an evidence-based doctor? Eur Urol. 2023;84(3):355-356. doi:10.1016/j.eururo.2023.03.037 37061445

[152] Zhou J, He X, Sun L, . SkinGPT-4: an interactive dermatology diagnostic system with visual large language model. Published online April 20, 2023. MedRxiv.

[153] Zhou Y, Moon C, Szatkowski J, Moore D, Stevens J. Evaluating ChatGPT responses in the context of a 53-year-old male with a femoral neck fracture: a qualitative analysis. Eur J Orthop Surg Traumatol. 2024;34(2):927-955. doi:10.1007/s00590-023-03742-4 37776392 PMC10858115

[154] Zhu L, Mou W, Chen R. Can the ChatGPT and other large language models with internet-connected database solve the questions and concerns of patient with prostate cancer and help democratize medical knowledge? J Transl Med. 2023;21(1):269. doi:10.1186/s12967-023-04123-5 37076876 PMC10115367

[155] Zúñiga Salazar G, Zúñiga D, Vindel CL, . Efficacy of AI chats to determine an emergency: a comparison between OpenAI’s ChatGPT, Google Bard, and Microsoft Bing AI Chat. Cureus. 2023;15(9):e45473. doi:10.7759/cureus.45473 37727841 PMC10506659

[156] Glasziou P, Meats E, Heneghan C, Shepperd S. What is missing from descriptions of treatment in trials and reviews? BMJ. 2008;336(7659):1472-1474. doi:10.1136/bmj.39590.732037.47 18583680 PMC2440840

[157] Wang C, Liu SX, Awadallah AH. Cost-effective hyperparameter optimization for large language model generation inference. Preprint published online August 8, 2023. arXiv. doi:10.48550/arXiv.2303.04673

[158] Wang PH, Hsieh SI, Chang SC, . Contextual temperature for language modeling. Published online December 25, 2020. arXiv. doi:10.48550/arXiv.2012.12575

[159] Wang R, Wang H, Mi F, . Enhancing Large language models against inductive instructions with dual-critique prompting. Published online March 7, 2024. arXiv. doi:10.48550/arXiv.2305.13733

[160] Meskó B. Prompt engineering as an important emerging skill for medical professionals: tutorial. J Med Internet Res. 2023;25(1):e50638. doi:10.2196/50638 37792434 PMC10585440

[161] Nguyen D, Swanson D, Newbury A, Kim YH. Evaluation of ChatGPT and Google Bard using prompt engineering in cancer screening algorithms. Acad Radiol. 2024;31(5):1799-1804. doi:10.1016/j.acra.2023.11.002 38103973

[162] Tian S, Jin Q, Yeganova L, . Opportunities and challenges for ChatGPT and large language models in biomedicine and health. Brief Bioinform. 2023;25(1):1-13. doi:10.1093/bib/bbad493 38168838 PMC10762511

[163] Russe MF, Reisert M, Bamberg F, Rau A. Improving the use of LLMs in radiology through prompt engineering: from precision prompts to zero-shot learning. Rofo. 2024;196(11):1166-1170. doi:10.1055/a-2264-5631 38408477

[164] Alowais SA, Alghamdi SS, Alsuhebany N, . Revolutionizing healthcare: the role of artificial intelligence in clinical practice. BMC Med Educ. 2023;23(1):689. doi:10.1186/s12909-023-04698-z 37740191 PMC10517477

[165] Guyatt GH, Oxman AD, Kunz R, . GRADE guidelines: 2. framing the question and deciding on important outcomes. J Clin Epidemiol. 2011;64(4):395-400. doi:10.1016/j.jclinepi.2010.09.012 21194891

[166] Chang Y, Wang X, Wang J, . A survey on evaluation of large language models. ACM Trans Intell Syst Technol. 2024;15(3):1-45. doi:10.1145/3641289

[167] Liang JJ, Tsou CH, Devarakonda MV. Ground truth creation for complex clinical NLP tasks: an iterative vetting approach and lessons learned. AMIA Jt Summits Transl Sci Proc. 2017;2017:203-212. 28815130 PMC5543376

[168] Ahmed W, Saturno M, Rajjoub R, . ChatGPT versus NASS clinical guidelines for degenerative spondylolisthesis: a comparative analysis. Eur Spine J. 2024;33(11):4182-4203. doi:10.1007/s00586-024-08198-6 38489044

[169] Gianola S, Bargeri S, Castellini G, . Performance of ChatGPT compared to clinical practice guidelines in making informed decisions for lumbosacral radicular pain: a cross-sectional study. J Orthop Sports Phys Ther. 2024;54(3):222-228. doi:10.2519/jospt.2024.12151 38284363

[170] Da Silva M, Flood CM, Goldenberg A, Singh D. Regulating the safety of health–related artificial intelligence. Healthc Policy. 2022;17(4):63-77. doi:10.12927/hcpol.2022.26824 35686827 PMC9170055

[171] FDA. Artificial intelligence and machine learning in software as a medical device. 2024. Accessed March 29, 2024. https://www.fda.gov/medical-devices/software-medical-device-samd/artificialintelligence-and-machine-learning-software-medical-device

[172] Health Canada. Guidance document: software as a medical device (SaMD): classification examples. 2022. Accessed March 29, 2024. https://www.canada.ca/en/health-canada/services/drugs-health-products/medical-devices/application-information/guidance-documents/software medical-device-guidance/examples.html#a4.3

[173] FDA. Artificial intelligence and machine learning (AI/ML)–enabled medical devices. Accessed March 29, 2024. https://www.fda.gov/medical-devices/software-medical-device-samd/artificial-intelligence-and-machine-learning-aiml-enabled-medical-devices

[174] Wornow M, Xu Y, Thapa R, . The shaky foundations of large language models and foundation models for electronic health records. NPJ Digit Med. 2023;6(1):135. doi:10.1038/s41746-023-00879-8 37516790 PMC10387101

[175] Microsoft. Microsoft and Epic expand strategic collaboration with integration of Azure OpenAI Service. Accessed March 29, 2024. https://news.microsoft.com/2023/04/17/microsoft-and-epic-expand-strategic-collaboration-with-integration-of-azure-openai-service/

[176] Dahdah R. Microsoft makes the promise of AI in healthcare real through new collaborations with healthcare organizations and partners. Accessed March 28, 2024. https://blogs.microsoft.com/blog/2024/03/11/microsoft-makes-the-promise-of-ai-in-healthcare-real-through-new-collaborations-with-healthcare-organizations-and-partners/

[177] Bitkina OV, Park J, Kim HK. Application of artificial intelligence in medical technologies: a systematic review of main trends. Digit Health. 2023;9:20552076231189331. doi:10.1177/20552076231189331 37485326 PMC10359663

[178] Saenz AD, Harned Z, Banerjee O, Abràmoff MD, Rajpurkar P. Autonomous AI systems in the face of liability, regulations and costs. NPJ Digit Med. 2023;6(1):185. doi:10.1038/s41746-023-00929-1 37803209 PMC10558567

[179] Wu E, Wu K, Daneshjou R, Ouyang D, Ho DE, Zou J. How medical AI devices are evaluated: limitations and recommendations from an analysis of FDA approvals. Nat Med. 2021;27(4):582-584. doi:10.1038/s41591-021-01312-x 33820998

[180] Nagendran M, Chen Y, Lovejoy CA, . Artificial intelligence versus clinicians: systematic review of design, reporting standards, and claims of deep learning studies in medical imaging. BMJ. 2020;368:m689. doi:10.1136/bmj.m689 PMC7190037 32213531

[181] Ong JCL, Chang SYH, William W, . Ethical and regulatory challenges of large language models in medicine. Lancet Digit Health. 2024;6(6):e428-e432. doi:10.1016/S2589-7500(24)00061-X 38658283

[182] World Health Organization. Ethics and governance of artificial intelligence for health. Accessed November 4, 2024. https://www.who.int/publications/i/item/9789240029200

[183] Thirunavukarasu AJ. Large language models will not replace healthcare professionals: curbing popular fears and hype. J R Soc Med. 2023;116(5):181-182. doi:10.1177/01410768231173123 37199678 PMC10331084

[184] Qu Y, Wang J. Performance and biases of large language models in public opinion simulation. Humanit Soc Sci Commun. 2024;11(1). doi:10.1057/s41599-024-03609-x

[185] Omiye JA, Lester JC, Spichak S, Rotemberg V, Daneshjou R. Large language models propagate race-based medicine. NPJ Digit Med. 2023;6(1):195. doi:10.1038/s41746-023-00939-z 37864012 PMC10589311

[186] Ayoub NF, Balakrishnan K, Ayoub MS, Barrett TF, David AP, Gray ST. Inherent bias in large language models: a random sampling analysis. Mayo Clin Proc Digit Health. 2024;2(2):186-191. doi:10.1016/j.mcpdig.2024.03.003 PMC11975844 40207170

[187] Huo B, Calabrese E, Sylla P, . The performance of artificial intelligence large language model–linked chatbots in surgical decision-making for gastroesophageal reflux disease. Surg Endosc. 2024;38(5):2320-2330. doi:10.1007/s00464-024-10807-w 38630178

[188] Au Yeung J, Kraljevic Z, Luintel A, . AI chatbots not yet ready for clinical use. Front Digit Health. 2023;5:1161098. doi:10.3389/fdgth.2023.1161098 37122812 PMC10130576

[189] Meyrowitsch DW, Jensen AK, Sørensen JB, Varga TV. AI chatbots and (mis)information in public health: impact on vulnerable communities. Front Public Health. 2023;11:1226776. doi:10.3389/fpubh.2023.1226776 38026315 PMC10644115

[190] Khan B, Fatima H, Qureshi A, . Drawbacks of artificial intelligence and their potential solutions in the healthcare sector. Biomed Mater Devices. 2023;1(2):1-8. doi:10.1007/s44174-023-00063-2 36785697 PMC9908503

[191] Yao Y, Duan J, Xu K, Cai Y, Sun Z, Zhang Y. A survey on large language model (LLM) security and privacy: the good, the bad, and the ugly. High-Confidence Computing. 2024;4(2). doi:10.1016/j.hcc.2024.100211

[192] Wang C, Liu S, Yang H, Guo J, Wu Y, Liu J. Ethical considerations of using ChatGPT in health care. J Med Internet Res. 2023;25:e48009. doi:10.2196/48009 37566454 PMC10457697

[193] Luo H, Specia L. From understanding to utilization: a survey on explainability for large language models. arXiv. Preprint posted online January 23, 2024. doi:10.48550/arXiv/2401.12874

