# Error in Author Degree

**DOI:** 10.1001/jamanetworkopen.2025.6511

**Published:** 2025-03-21

In the Original Investigation titled “Large Language Models for Chatbot Health Advice Studies: A Systematic Review,”^1^ published February 4, 2025, there was an error in the degree given for fourth author Winmonchat Tangamornsuksan. The degree given as “BSc” should have been “PharmD, PhD.” This article has been corrected.^1^
