# Supplementary material for: Large Language Models for Chatbot Health Advice Studies: A Systematic Review
Source: JAMA Netw Open. 2025 Feb 4;8(2):e2457879. doi: 10.1001/jamanetworkopen.2024.57879 (PMC11795331; doi:10.1001/jamanetworkopen.2024.57879)
Supplement: Supplement 1. — eAppendix. Literature Search Syntax [file jamanetwopen-e2457879-s001.pdf]

Supplementary Online Content

Huo B, Boyle A, Marfo N, et al. Large language models for chatbot health advice studies: a systematic review. *JAMA Netw Open*. 2025;8(2):e2457879.
doi:10.1001/jamanetworkopen.2024.57879

eAppendix. Literature Search Syntax

This supplementary material has been provided by the authors to give readers additional information about their work.

© 2025 Huo B et al. *JAMA Network Open.*

eAppendix 1. Literature Search Syntax.

Ovid MEDLINE(R) ALL <1946 to October 25, 2023>

1	generative ai.ti,ab,kf.	153
2	generative artificial intelligence.ti,ab,kf.	124
3	AI-based chatbots.ti,ab,kf.	8
4	chatgpt.ti,ab,kf.	1525
5	bing chat.ti,ab,kf.	5
6	google bard.ti,ab,kf.	26
7	chatbot.ti,ab,kf.	929
8	large language model.ti,ab,kf.	245
9	chatgpt-4.ti,ab,kf.	65
10	gpt-4.ti,ab,kf.	157
11	natural language processing/	6244
12	natural language processing.ti,ab,kf.	8365
13	LLM.ti,ab,kf.	462
14	NLP.ti,ab,kf.	4260
15	("concept extraction" or "concept recognition" or "extraction framework" or "information extraction" or "medical language processing" or "named entity recognition" or "natural language processing" or "symptom extraction" or "text mining").ti,ab,kf.	13661
16	1 or 2 or 8 or 11 or 12 or 13 or 14	13339
17	7 and 16	176
18	4 or 5 or 6 or 9 or 10	1602
19	17 or 18	1680
20	(clinical adj2 (advice* or decision-making* or knowledge* or questions* or recommendations* or decision support* or assessment* or information*)).ti,ab,kf.	145267
21	(expert adj2 (advice* or decision-making* or knowledge* or questions* or recommendations* or decision support* or assessment* or information*)).ti,ab,kf.	8749
22	(patient adj2 (advice* or decision-making* or knowledge* or questions* or recommendations* or decision support* or assessment* or information*)).ti,ab,kf.	44226
23	(surgical adj2 (advice* or decision-making* or knowledge* or questions* or recommendations* or decision support* or assessment* or information*)).ti,ab,kf.	10801
24	(medical adj2 (advice* or decision-making* or knowledge* or questions* or recommendations* or decision support* or assessment* or information*)).ti,ab,kf.	43590
25	(health adj2 (advice* or decision-making* or knowledge* or questions* or recommendations* or decision support* or assessment* or information*)).ti,ab,kf.	102275
26	(screening adj2 (advice* or decision-making* or knowledge* or questions* or recommendations* or decision support* or assessment* or information*)).ti,ab,kf.	11818
27	(health prevention adj2 (advice* or decision-making* or knowledge* or questions* or recommendations* or decision support* or assessment* or information*)).ti,ab,kf.	13
28	(diagnos* adj2 (advice* or decision-making* or knowledge* or questions* or recommendations* or decision support* or assessment* or information*)).ti,ab,kf.	34544
29	(differential diagnos* adj2 (advice* or decision-making* or knowledge* or questions* or recommendations* or decision support* or assessment* or information*)).ti,ab,kf.	739
30	(treatment adj2 (advice* or decision-making* or knowledge* or questions* or recommendations* or decision support* or assessment* or information*)).ti,ab,kf.	48632
31	(management adj2 (advice* or decision-making* or knowledge* or questions* or recommendations* or decision support* or assessment* or information*)).ti,ab,kf.	34007
32	20 or 21 or 22 or 23 or 24 or 25 or 26 or 27 or 28 or 29 or 30 or 31	445717
33	19 and 32	292
34	(chatbot adj3 (LLM* or large language model* or NLP* or natural language processing* or AI* or artificial intelligence* or generative*)).ti,ab,kf.	230
35	17 or 18 or 34	1767
36	32 and 35	304
37	1 or 2 or 3 or 4 or 5 or 6 or 7 or 8 or 9 or 10 or 11 or 12 or 13 or 14 or 15 or 34	19918
38	32 and 37	2573

© 2025 Huo B et al. *JAMA Network Open.*

Embase <1974 to 2023 October 25>

1	generative ai.ti,ab,kf.	135
2	generative artificial intelligence.ti,ab,kf.	100
3	AI-based chatbots.ti,ab,kf.	9
4	chatgpt.ti,ab,kf.	1368
5	bing chat.ti,ab,kf.	5
6	google bard.ti,ab,kf.	22
7	chatbot.ti,ab,kf.	892
8	large language model.ti,ab,kf.	228
9	chatgpt-4.ti,ab,kf.	53
10	gpt-4.ti,ab,kf.	184
11	natural language processing/	10814
12	natural language processing.ti,ab,kf.	9634
13	LLM.ti,ab,kf.	606
14	NLP.ti,ab,kf.	5238
15	("concept extraction" or "concept recognition" or "extraction framework" or "information extraction" or "medical language processing" or "named entity recognition" or "natural language processing" or "symptom extraction" or "text mining").ti,ab,kf.	15186
16	1 or 2 or 8 or 11 or 12 or 13 or 14	15565
17	7 and 16	167
18	4 or 5 or 6 or 9 or 10	1455
19	17 or 18	1539
20	(clinical adj2 (advice* or decision-making* or knowledge* or questions* or recommendations* or decision support* or assessment* or information*)).ti,ab,kf.	217997
21	(expert adj2 (advice* or decision-making* or knowledge* or questions* or recommendations* or decision support* or assessment* or information*)).ti,ab,kf.	11464
22	(patient adj2 (advice* or decision-making* or knowledge* or questions* or recommendations* or decision support* or assessment* or information*)).ti,ab,kf.	75234
23	(surgical adj2 (advice* or decision-making* or knowledge* or questions* or recommendations* or decision support* or assessment* or information*)).ti,ab,kf.	15334
24	(medical adj2 (advice* or decision-making* or knowledge* or questions* or recommendations* or decision support* or assessment* or information*)).ti,ab,kf.	61444
25	(health adj2 (advice* or decision-making* or knowledge* or questions* or recommendations* or decision support* or assessment* or information*)).ti,ab,kf.	131219
26	(screening adj2 (advice* or decision-making* or knowledge* or questions* or recommendations* or decision support* or assessment* or information*)).ti,ab,kf.	17383
27	(health prevention adj2 (advice* or decision-making* or knowledge* or questions* or recommendations* or decision support* or assessment* or information*)).ti,ab,kf.	24
28	(diagnos* adj2 (advice* or decision-making* or knowledge* or questions* or recommendations* or decision support* or assessment* or information*)).ti,ab,kf.	48996
29	(differential diagnos* adj2 (advice* or decision-making* or knowledge* or questions* or recommendations* or decision support* or assessment* or information*)).ti,ab,kf.	1084
30	(treatment adj2 (advice* or decision-making* or knowledge* or questions* or recommendations* or decision support* or assessment* or information*)).ti,ab,kf.	75332
31	(management adj2 (advice* or decision-making* or knowledge* or questions* or recommendations* or decision support* or assessment* or information*)).ti,ab,kf.	46641
32	20 or 21 or 22 or 23 or 24 or 25 or 26 or 27 or 28 or 29 or 30 or 31	643009
33	19 and 32	292
34	(chatbot adj3 (LLM* or large language model* or NLP* or natural language processing* or AI* or artificial intelligence* or generative*)).ti,ab,kf.	214
35	17 or 18 or 34	1632
36	32 and 35	307
37	1 or 2 or 3 or 4 or 5 or 6 or 7 or 8 or 9 or 10 or 11 or 12 or 13 or 14 or 15 or 34	22375
38	32 and 37	2867

© 2025 Huo B et al. *JAMA Network Open.*

Web of Science Search Strategy (v0.1)

Database: Web of Science Core Collection

Entitlements:

- WOS.IC: 1993 to 2023
- WOS.CCR: 1985 to 2023
- WOS.SCI: 1976 to 2023
- WOS.AHCI: 1976 to 2023
- WOS.BHCI: 2005 to 2023
- WOS.BSCI: 2005 to 2023
- WOS.ESCI: 2018 to 2023
- WOS.ISTP: 1990 to 2023
- WOS.SSCI: 1976 to 2023
- WOS.ISSHP: 1990 to 2023

Searches:

1: ALL=("generative artificial intelligence") Date Run: Thu Oct 26 2023 20:42:35 GMT-0400 (Eastern Daylight Time) Results: 212

2: ALL=("generative ai") Date Run: Thu Oct 26 2023 20:44:18 GMT-0400 (Eastern Daylight Time) Results: 304

3: ALL=("AI-based chatbots") Date Run: Thu Oct 26 2023 20:44:56 GMT-0400 (Eastern Daylight Time) Results: 27

4: ALL=("chatgpt") Date Run: Thu Oct 26 2023 20:45:07 GMT-0400 (Eastern Daylight Time) Results: 1673

5: ALL=("bing chat") Date Run: Thu Oct 26 2023 20:45:20 GMT-0400 (Eastern Daylight Time) Results: 2

6: ALL=("google bard") Date Run: Thu Oct 26 2023 20:45:28 GMT-0400 (Eastern Daylight Time) Results: 15

7: ALL=("chatbot") Date Run: Thu Oct 26 2023 20:45:38 GMT-0400 (Eastern Daylight Time) Results: 3292

8: ALL=("large language model") Date Run: Thu Oct 26 2023 20:46:15 GMT-0400 (Eastern Daylight Time) Results: 252

9: ALL=("chatgpt-4") Date Run: Thu Oct 26 2023 20:46:28 GMT-0400 (Eastern Daylight Time) Results: 58

10: ALL=("gpt-4") Date Run: Thu Oct 26 2023 20:46:37 GMT-0400 (Eastern Daylight Time) Results: 139

11: ALL=("natural language processing") Date Run: Thu Oct 26 2023 20:46:53 GMT-0400 (Eastern Daylight Time) Results: 44481

© 2025 Huo B et al. *JAMA Network Open.*

12: ALL=("LLM") Date Run: Thu Oct 26 2023 20:47:08 GMT-0400 (Eastern Daylight Time) Results: 3455

13: ALL=("NLP") Date Run: Thu Oct 26 2023 20:47:14 GMT-0400 (Eastern Daylight Time) Results: 18868

14: ALL=("concept extraction" or "concept recognition" or "extraction framework" or "information extraction" or "medical language processing" or "named entity recognition" or "natural language processing" or "symptom extraction" or "text mining") Date Run: Thu Oct 26 2023 20:47:52 GMT-0400 (Eastern Daylight Time) Results: 73332

15: TS = (chatbot NEAR/5 "llm") Date Run: Thu Oct 26 2023 21:12:35 GMT-0400 (Eastern Daylight Time) Results: 2

16: TS = (chatbot NEAR/5 "large language model") Date Run: Thu Oct 26 2023 21:15:58 GMT-0400 (Eastern Daylight Time) Results: 9

17: TS = (chatbot NEAR/5 "nlp") Date Run: Thu Oct 26 2023 21:18:14 GMT-0400 (Eastern Daylight Time) Results: 19

18: TS = (chatbot NEAR/5 "natural language processing") Date Run: Thu Oct 26 2023 21:18:22 GMT-0400 (Eastern Daylight Time) Results: 47

19: TS = (chatbot NEAR/5 "AI") Date Run: Thu Oct 26 2023 21:18:36 GMT-0400 (Eastern Daylight Time) Results: 281

20: TS = (chatbot NEAR/5 "artificial intelligence") Date Run: Thu Oct 26 2023 21:18:46 GMT-0400 (Eastern Daylight Time) Results: 210

21: TS = (chatbot NEAR/5 "large language model") Date Run: Thu Oct 26 2023 21:19:01 GMT-0400 (Eastern Daylight Time) Results: 9

22: #21 OR #20 OR #19 OR #18 OR #17 OR #16 OR #15 OR #14 OR #13 OR #12 OR #11 OR #10 OR #9 OR #8 OR #7 OR #6 OR #5 OR #4 OR #3 OR #2 OR #1 Date Run: Thu Oct 26 2023 21:20:14 GMT-0400 (Eastern Daylight Time) Results: 90334

23: ALL=("clinical advice") Date Run: Thu Oct 26 2023 21:21:11 GMT-0400 (Eastern Daylight Time) Results: 537

24: ALL=("clinical decision-making") Date Run: Thu Oct 26 2023 21:21:20 GMT-0400 (Eastern Daylight Time) Results: 26753

25: ALL=("clinical knowledge") Date Run: Thu Oct 26 2023 21:21:27 GMT-0400 (Eastern Daylight Time) Results: 3490

26: ALL=("clinical questions") Date Run: Thu Oct 26 2023 21:21:32 GMT-0400 (Eastern Daylight Time) Results: 3748

27: ALL=("clinical recommendations") Date Run: Thu Oct 26 2023 21:21:40 GMT-0400 (Eastern Daylight Time) Results: 4074

28: ALL=("clinical decision support") Date Run: Thu Oct 26 2023 21:21:44 GMT-0400 (Eastern Daylight Time) Results: 10518

29: ALL=("clinical assessment") Date Run: Thu Oct 26 2023 21:21:50 GMT-0400 (Eastern Daylight Time) Results: 30582

© 2025 Huo B et al. *JAMA Network Open.*

30: ALL=("clinical information") Date Run: Thu Oct 26 2023 21:21:53 GMT-0400 (Eastern Daylight Time) Results: 25941

31: ALL=("expert advice") Date Run: Thu Oct 26 2023 21:22:04 GMT-0400 (Eastern Daylight Time) Results: 5934

32: ALL=("expert decision-making") Date Run: Thu Oct 26 2023 21:22:09 GMT-0400 (Eastern Daylight Time) Results: 350

33: ALL=("expert knowledge") Date Run: Thu Oct 26 2023 21:22:16 GMT-0400 (Eastern Daylight Time) Results: 13182

34: ALL=("expert questions") Date Run: Thu Oct 26 2023 21:22:21 GMT-0400 (Eastern Daylight Time) Results: 15

35: ALL=("expert recommendations") Date Run: Thu Oct 26 2023 21:22:27 GMT-0400 (Eastern Daylight Time) Results: 1402

36: ALL=("expert decision support") Date Run: Thu Oct 26 2023 21:22:33 GMT-0400 (Eastern Daylight Time) Results: 106

37: ALL=("expert assessment") Date Run: Thu Oct 26 2023 21:22:40 GMT-0400 (Eastern Daylight Time) Results: 1665

38: ALL=("expert information") Date Run: Thu Oct 26 2023 21:22:44 GMT-0400 (Eastern Daylight Time) Results: 581

39: ALL=("patient advice") Date Run: Thu Oct 26 2023 21:22:50 GMT-0400 (Eastern Daylight Time) Results: 150

40: ALL=("patient decision-making") Date Run: Thu Oct 26 2023 21:22:53 GMT-0400 (Eastern Daylight Time) Results: 1099

41: ALL=("patient knowledge") Date Run: Thu Oct 26 2023 21:23:02 GMT-0400 (Eastern Daylight Time) Results: 2340

42: ALL=("patient questions") Date Run: Thu Oct 26 2023 21:23:05 GMT-0400 (Eastern Daylight Time) Results: 249

43: ALL=("patient recommendations") Date Run: Thu Oct 26 2023 21:23:08 GMT-0400 (Eastern Daylight Time) Results: 111

44: ALL=("patient decision support") Date Run: Thu Oct 26 2023 21:23:12 GMT-0400 (Eastern Daylight Time) Results: 92

45: ALL=("patient assessment") Date Run: Thu Oct 26 2023 21:23:16 GMT-0400 (Eastern Daylight Time) Results: 4123

46: ALL=("patient information") Date Run: Thu Oct 26 2023 21:23:19 GMT-0400 (Eastern Daylight Time) Results: 10627

47: ALL=("surgical advice") Date Run: Thu Oct 26 2023 21:23:26 GMT-0400 (Eastern Daylight Time) Results: 44

© 2025 Huo B et al. *JAMA Network Open.*

48: ALL=("medical advice") Date Run: Thu Oct 26 2023 21:23:31 GMT-0400 (Eastern Daylight Time) Results: 4424

49: ALL=("surgical decision-making") Date Run: Thu Oct 26 2023 21:23:36 GMT-0400 (Eastern Daylight Time) Results: 3188

50: ALL=("medical decision-making") Date Run: Thu Oct 26 2023 21:23:38 GMT-0400 (Eastern Daylight Time) Results: 10476

51: ALL=("surgical knowledge") Date Run: Thu Oct 26 2023 21:23:47 GMT-0400 (Eastern Daylight Time) Results: 307

52: ALL=("medical knowledge") Date Run: Thu Oct 26 2023 21:23:50 GMT-0400 (Eastern Daylight Time) Results: 6943

53: ALL=("surgical questions") Date Run: Thu Oct 26 2023 21:23:57 GMT-0400 (Eastern Daylight Time) Results: 31

54: ALL=("medical questions") Date Run: Thu Oct 26 2023 21:24:01 GMT-0400 (Eastern Daylight Time) Results: 310

55: ALL=("surgical recommendations") Date Run: Thu Oct 26 2023 21:24:07 GMT-0400 (Eastern Daylight Time) Results: 157

56: ALL=("medical recommendations") Date Run: Thu Oct 26 2023 21:24:09 GMT-0400 (Eastern Daylight Time) Results: 615

57: ALL=("surgical decision support") Date Run: Thu Oct 26 2023 21:24:15 GMT-0400 (Eastern Daylight Time) Results: 13

58: ALL=("medical decision support") Date Run: Thu Oct 26 2023 21:24:17 GMT-0400 (Eastern Daylight Time) Results: 865

59: ALL=("surgical assessment") Date Run: Thu Oct 26 2023 21:24:24 GMT-0400 (Eastern Daylight Time) Results: 742

60: ALL=("medical assessment") Date Run: Thu Oct 26 2023 21:24:26 GMT-0400 (Eastern Daylight Time) Results: 1644

61: ALL=("surgical information") Date Run: Thu Oct 26 2023 21:24:34 GMT-0400 (Eastern Daylight Time) Results: 426

62: ALL=("medical information") Date Run: Thu Oct 26 2023 21:24:39 GMT-0400 (Eastern Daylight Time) Results: 14223

63: ALL=("health advice") Date Run: Thu Oct 26 2023 21:24:45 GMT-0400 (Eastern Daylight Time) Results: 1306

64: ALL=("health decision-making") Date Run: Thu Oct 26 2023 21:24:50 GMT-0400 (Eastern Daylight Time) Results: 1485

65: ALL=("health knowledge") Date Run: Thu Oct 26 2023 21:24:56 GMT-0400 (Eastern Daylight Time) Results: 5952

66: ALL=("health questions") Date Run: Thu Oct 26 2023 21:24:59 GMT-0400 (Eastern Daylight Time) Results: 610

© 2025 Huo B et al. *JAMA Network Open.*

67: ALL=("health recommendations") Date Run: Thu Oct 26 2023 21:25:02 GMT-0400 (Eastern Daylight Time) Results: 1638

68: ALL=("health decision support") Date Run: Thu Oct 26 2023 21:25:06 GMT-0400 (Eastern Daylight Time) Results: 90

69: ALL=("health assessment") Date Run: Thu Oct 26 2023 21:25:10 GMT-0400 (Eastern Daylight Time) Results: 15041

70: ALL=("health information") Date Run: Thu Oct 26 2023 21:25:18 GMT-0400 (Eastern Daylight Time) Results: 41464

71: ALL=("screening advice") Date Run: Thu Oct 26 2023 21:25:41 GMT-0400 (Eastern Daylight Time) Results: 43

72: ALL=("screening decision-making") Date Run: Thu Oct 26 2023 21:25:46 GMT-0400 (Eastern Daylight Time) Results: 99

73: ALL=("screening knowledge") Date Run: Thu Oct 26 2023 21:25:50 GMT-0400 (Eastern Daylight Time) Results: 353

74: ALL=("screening questions") Date Run: Thu Oct 26 2023 21:25:54 GMT-0400 (Eastern Daylight Time) Results: 1071

75: ALL=("screening recommendations") Date Run: Thu Oct 26 2023 21:25:59 GMT-0400 (Eastern Daylight Time) Results: 1939

76: ALL=("screening decision support") Date Run: Thu Oct 26 2023 21:26:05 GMT-0400 (Eastern Daylight Time) Results: 11

77: ALL=("screening assessment") Date Run: Thu Oct 26 2023 21:26:09 GMT-0400 (Eastern Daylight Time) Results: 1367

78: ALL=("screening information") Date Run: Thu Oct 26 2023 21:26:13 GMT-0400 (Eastern Daylight Time) Results: 460

79: ALL=("health prevention advice") Date Run: Thu Oct 26 2023 21:26:21 GMT-0400 (Eastern Daylight Time) Results: 2

80: ALL=("health prevention decision-making") Date Run: Thu Oct 26 2023 21:26:27 GMT-0400 (Eastern Daylight Time) Results: 0

81: ALL=("health prevention knowledge") Date Run: Thu Oct 26 2023 21:26:33 GMT-0400 (Eastern Daylight Time) Results: 1

82: ALL=("health prevention questions") Date Run: Thu Oct 26 2023 21:26:37 GMT-0400 (Eastern Daylight Time) Results: 0

83: ALL=("health prevention recommendations") Date Run: Thu Oct 26 2023 21:26:40 GMT-0400 (Eastern Daylight Time) Results: 0

84: ALL=("health prevention decision support") Date Run: Thu Oct 26 2023 21:26:44 GMT-0400 (Eastern Daylight Time) Results: 0

© 2025 Huo B et al. *JAMA Network Open.*

85: ALL=("health prevention assessment") Date Run: Thu Oct 26 2023 21:26:50 GMT-0400 (Eastern Daylight Time) Results: 1

86: ALL=("health prevention information") Date Run: Thu Oct 26 2023 21:26:54 GMT-0400 (Eastern Daylight Time) Results: 6

87: ALL=("diagnos* advice") Date Run: Thu Oct 26 2023 21:27:14 GMT-0400 (Eastern Daylight Time) Results: 104

88: ALL=("diagnos* decision-making") Date Run: Thu Oct 26 2023 21:27:48 GMT-0400 (Eastern Daylight Time) Results: 772

89: ALL=("diagnos* knowledge") Date Run: Thu Oct 26 2023 21:27:53 GMT-0400 (Eastern Daylight Time) Results: 805

90: ALL=("diagnos* questions") Date Run: Thu Oct 26 2023 21:27:57 GMT-0400 (Eastern Daylight Time) Results: 353

91: ALL=("diagnos* recommendations") Date Run: Thu Oct 26 2023 21:28:01 GMT-0400 (Eastern Daylight Time) Results: 311

92: ALL=("diagnos* decision support") Date Run: Thu Oct 26 2023 21:28:03 GMT-0400 (Eastern Daylight Time) Results: 337

93: ALL=("diagnos* assessment") Date Run: Thu Oct 26 2023 21:28:07 GMT-0400 (Eastern Daylight Time) Results: 5892

94: ALL=("diagnos* information") Date Run: Thu Oct 26 2023 21:28:10 GMT-0400 (Eastern Daylight Time) Results: 8805

95: ALL=("differential diagnos* advice") Date Run: Thu Oct 26 2023 21:28:18 GMT-0400 (Eastern Daylight Time) Results: 1

96: ALL=("differential diagnos* decision-making") Date Run: Thu Oct 26 2023 21:28:24 GMT-0400 (Eastern Daylight Time) Results: 5

97: ALL=("differential diagnos* knowledge") Date Run: Thu Oct 26 2023 21:28:30 GMT-0400 (Eastern Daylight Time) Results: 14

98: ALL=("differential diagnos* questions") Date Run: Thu Oct 26 2023 21:28:32 GMT-0400 (Eastern Daylight Time) Results: 9

99: ALL=("differential diagnos* recommendations") Date Run: Thu Oct 26 2023 21:28:40 GMT-0400 (Eastern Daylight Time) Results: 2

100: ALL=("differential diagnos* decision support") Date Run: Thu Oct 26 2023 21:28:52 GMT-0400 (Eastern Daylight Time) Results: 3

101: ALL=("differential diagnos* assessment") Date Run: Thu Oct 26 2023 21:28:57 GMT-0400 (Eastern Daylight Time) Results: 50

102: ALL=("differential diagnos* information") Date Run: Thu Oct 26 2023 21:29:09 GMT-0400 (Eastern Daylight Time) Results: 28

103: ALL=("treatment advice") Date Run: Thu Oct 26 2023 21:29:18 GMT-0400 (Eastern Daylight Time) Results: 355

© 2025 Huo B et al. *JAMA Network Open.*

104: ALL=("treatment decision-making") Date Run: Thu Oct 26 2023 21:29:23 GMT-0400 (Eastern Daylight Time) Results: 4882

105: ALL=("treatment knowledge") Date Run: Thu Oct 26 2023 21:29:27 GMT-0400 (Eastern Daylight Time) Results: 471

106: ALL=("treatment questions") Date Run: Thu Oct 26 2023 21:29:30 GMT-0400 (Eastern Daylight Time) Results: 158

107: ALL=("treatment recommendations") Date Run: Thu Oct 26 2023 21:29:33 GMT-0400 (Eastern Daylight Time) Results: 9650

108: ALL=("treatment decision support") Date Run: Thu Oct 26 2023 21:29:36 GMT-0400 (Eastern Daylight Time) Results: 92

109: ALL=("treatment assessment") Date Run: Thu Oct 26 2023 21:29:41 GMT-0400 (Eastern Daylight Time) Results: 1533

110: ALL=("treatment information") Date Run: Thu Oct 26 2023 21:29:43 GMT-0400 (Eastern Daylight Time) Results: 1807

111: ALL=("management advice") Date Run: Thu Oct 26 2023 21:29:47 GMT-0400 (Eastern Daylight Time) Results: 960

112: ALL=("management decision-making") Date Run: Thu Oct 26 2023 21:29:50 GMT-0400 (Eastern Daylight Time) Results: 2972

113: ALL=("management knowledge") Date Run: Thu Oct 26 2023 21:29:55 GMT-0400 (Eastern Daylight Time) Results: 2371

114: ALL=("management questions") Date Run: Thu Oct 26 2023 21:29:58 GMT-0400 (Eastern Daylight Time) Results: 580

115: ALL=("management recommendations") Date Run: Thu Oct 26 2023 21:30:01 GMT-0400 (Eastern Daylight Time) Results: 3690

116: ALL=("management decision support") Date Run: Thu Oct 26 2023 21:30:05 GMT-0400 (Eastern Daylight Time) Results: 673

117: ALL=("management assessment") Date Run: Thu Oct 26 2023 21:30:09 GMT-0400 (Eastern Daylight Time) Results: 969

118: ALL=("management information") Date Run: Thu Oct 26 2023 21:30:13 GMT-0400 (Eastern Daylight Time) Results: 13272

119: #118 OR #117 OR #116 OR #115 OR #114 OR #113 OR #112 OR #111 OR #110 OR #109 OR #108 OR #107 OR #106 OR #105 OR #104 OR #103 OR #102 OR #101 OR #100 OR #99 OR #98 OR #97 OR #96 OR #95 OR #94 OR #93 OR #92 OR #91 OR #90 OR #89 OR #88 OR #87 OR #86 OR #85 OR #84 OR #83 OR #82 OR #81 OR #80 OR #79 OR #78 OR #77 OR #76 OR #75 OR #74 OR #73 OR #72 OR #71 OR #70 OR #69 OR #68 OR #67 OR #66 OR #65 OR #64 OR #63 OR #62 OR #61 OR #60 OR #59 OR #58 OR #57 OR #56 OR #55 OR #54 OR #53 OR #52 OR #51 OR #50 OR #49 OR #48 OR #47 OR #46 OR #45 OR #44 OR #43 OR #42 OR #41 OR #40 OR #39 OR #38 OR #37 OR #36 OR #35 OR #34 OR #33 OR #32 OR #31 OR #30 OR #29 OR #28 OR #27 OR #26 OR #25 OR #24 OR #23 Date Run: Thu Oct 26 2023 21:30:48 GMT-0400 (Eastern Daylight Time) Results: 310222

© 2025 Huo B et al. *JAMA Network Open.*

120: #110 OR #107 OR #106 OR #103 OR #101 OR #100 OR #99 OR #98 OR #95 OR #93 OR #92 OR #91 OR #88 OR #87 OR #86 OR #78 OR #77 OR #75 OR #74 OR #71 OR #72 OR #70 OR #69 OR #67 OR #66 OR #65 OR #64 OR #63 OR #62 OR #61 OR #60 OR #59 OR #58 OR #56 OR #55 OR #57 OR #52 OR #51 OR #54 OR #53 OR #50 OR #49 OR #48 OR #47 OR #46 OR #45 OR #43 OR #41 OR #40 OR #39 OR #37 OR #35 OR #33 OR #31 OR #30 OR #29 OR #28 OR #27 OR #26 OR #25 OR #24 OR #23 Date Run: Thu Oct 26 2023 21:35:34 GMT-0400 (Eastern Daylight Time) Results: 268665

121: #120 AND #22 Date Run: Thu Oct 26 2023 21:36:00 GMT-0400 (Eastern Daylight Time) Results: 2306

© 2025 Huo B et al. *JAMA Network Open.*
