# Supplementary material for: Large Language Models for Chatbot Health Advice Studies: A Systematic Review
Source: JAMA Netw Open. 2025 Feb 4;8(2):e2457879. doi: 10.1001/jamanetworkopen.2024.57879 (PMC11795331; doi:10.1001/jamanetworkopen.2024.57879)
Supplement: Supplement 2. — Data Sharing Statement [file jamanetwopen-e2457879-s002.pdf]

Data Sharing Statement

Huo. Large Language Models for Chatbot Health Advice Studies. *JAMA Netw Open*. Published February 04, 2025. doi:10.1001/jamanetworkopen.2024.57879

Data
Data available: No

Additional Information
Explanation for why data not available: N/A. All data is being submitted in the manuscript.
